# Separate cortical and hippocampal cell populations target the rat nucleus reuniens and mammillary bodies

**DOI:** 10.1111/ejn.14341

**Published:** 2019-02-21

**Authors:** Mathias L. Mathiasen, Eman Amin, Andrew J. D. Nelson, Christopher M. Dillingham, Shane M. O'Mara, John P. Aggleton

**Affiliations:** ^1^ School of Psychology Cardiff University Wales UK; ^2^ Institute of Neuroscience Trinity College Dublin Ireland

**Keywords:** anatomy, frontal cortex, hippocampus, subiculum, thalamus

## Abstract

Nucleus reuniens receives dense projections from both the hippocampus and the frontal cortices. Reflecting these connections, this nucleus is thought to enable executive functions, including those involving spatial learning. The mammillary bodies, which also support spatial learning, again receive dense hippocampal inputs, as well as lighter projections from medial frontal areas. The present study, therefore, compared the sources of these inputs to nucleus reuniens and the mammillary bodies. Retrograde tracer injections in rats showed how these two diencephalic sites receive projections from separate cell populations, often from adjacent layers in the same cortical areas. In the subiculum, which projects strongly to both sites, the mammillary body inputs originate from a homogenous pyramidal cell population in more superficial levels, while the cells that target nucleus reuniens most often originate from cells positioned at a deeper level. In these deeper levels, a more morphologically diverse set of subiculum cells contributes to the thalamic projection, especially at septal levels. While both diencephalic sites also receive medial frontal inputs, those to nucleus reuniens are especially dense. The densest inputs to the mammillary bodies appear to arise from the dorsal peduncular cortex, where the cells are mostly separate from deeper neurons that project to nucleus reuniens. Again, in those other cortical regions that innervate both nucleus reuniens and the mammillary bodies, there was no evidence of collateral projections. The findings support the notion that these diencephalic nuclei represent components of distinct, but complementary, systems that support different aspects of cognition.

**Abbreviations**
3V: third ventricle
A11: A11 dopamine cells
A13: A13 dopamine cells
AcbC: nucleus accumbens core
AcbSh: nucleus accumbens shell
AHa: amygdalohippocampal area
AI: agranular insular cortex
AM: anteromedial thalamic nucleus
AV: anteroventral thalamic nucleus
CA: cornu ammonis
CB: cingulum bundle
CC: corpus callosum
Cg: anterior cingulate cortex
CM: central medial thalamic nucleus
Cpu: caudate putamen
CTB: cholera toxin b
DA: dorsal hypothalamic areas
DI: dysgranular insular cortex
DMC: dorsomedial hypothalamic nucleus compact part
DMD: dorsomedial hypothalamic nucleus dorsal part
DMV: dorsomedial hypothalamic nucleus ventral part
DP: dorsal peduncular cortex
dSUB: dorsal subiculum
FB: fast blue
fr: fasciculus retroflexus
IAM: interanteromedial thalamic nucleus
IG: indusium griseum
IL: infralimbic cortex
IMD: intermediodorsal thalamic nucleus
iSUB: intermediate subiculum
LM: lateral mammillary nucleus
LO: lateral orbital cortex
LSS: lateral septal nucleus dorsal part
M1: secondary motor cortex
M2: secondary motor cortex
MEC: medial entorhinal cortex
Me: median portion of the medial mammillary nucleus
ML: lateral portion of the medial mammillary nucleus
MM: medial portion of the medial mammillary nucleus
MO: medial orbital cortex
mp: mammillary peduncle
mt: mammillothalamic tract
PaS: parasubiculum
PaXi: paraxiphoid thalamic nucleus
PHD: posterior hypothalamic areas dorsal part
PH: posterior hypothalamic nucleus
PL: prelimbic cortex
POS: postsubiculum
PrS: presubiculum
PVT: paraventricular thalamic nucleus
RE: nucleus reuniens
Rh: rhomboid nucleus
RLi: rostral linear nucleus of the raphe
RSC: retrosplenial cortex
Rt: reticular thalamic nucleus
SMT: submedius thalamic nucleus
SUB: subiculum
SUM: supramammillary nucleus
TT: tenia tecta
VL: ventrolateral thalamic nucleus
VM: ventromedial thalamic nucleus
VO: ventral orbital cortex
VPM: ventral posteromedial thalamic nucleus
VPPC: ventral posterior nucleus of the thalamus parvicellular part
vRE: ventral nucleus reuniens
vSUB: ventral subiculum
ZIR: zona incerta rostral part
ZI: zona incerta

## 1 INTRODUCTION

Two diencephalic nuclei, the mammillary bodies (MB) and nucleus reuniens (RE) share anatomical and functional properties. Both nuclei receive dense inputs from the hippocampus, which include many projections from the subiculum (Allen & Hopkins, 1989; Herkenham, 1978; McKenna & Vertes, 2004; Meibach & Siegel, 1977). Both nuclei also receive frontal inputs (Allen & Hopkins, 1989; Herkenham, 1978; Hurley, Herbert, Moga, & Saper, 1991; McKenna & Vertes, 2004; Shibata, 1989) making them sites of fronto‐hippocampal information integration (Ito, Zhang, Witter, Moser, & Moser, 2015; Xu & Sudhof, 2013). In addition, electrophysiological recordings reveal that both the mammillary bodies and nucleus reuniens contain units with spatial properties, including head direction cells (Jankowski et al., 2014; Stackman & Taube, 1998). Consistent with these anatomical and electrophysiological findings, lesions in both sites can disrupt tests of spatial working memory such as location nonmatching (Beracochea & Jaffard, 1987; Cholvin et al., 2013; Hembrook & Mair, 2011; Hembrook, Onos, & Mair, 2012; Layfield, Patel, Hallock, & Griffin, 2015; Vann & Aggleton, 2003; Vann & Nelson, 2015). In addition, mammillary body lesions impair escape learning in a Morris water maze (Vann & Aggleton, 2003), in which animals locate a submerged platform. While lesions of nucleus reuniens spare initial acquisition of this same task, they affect performance during probe tests (Dolleman‐van der Weel, Morris, & Witter, 2009) and disrupt long‐term retention of the escape location (Loureiro et al., 2012). These patterns of anatomical and behavioural findings raise questions over the extent to which these two nuclei receive inputs from the same or different sources within the hippocampus and frontal cortices. Here, the term ‘frontal cortices’ incorporates infralimbic, prelimbic, anterior cingulate, precentral, orbital and agranular insular cortices (Kolb, 1984; Krettek & Price, 1977), although the terminology for sites within this region adheres to Paxinos and Watson (2004).

To determine whether the frontal and hippocampal inputs to these diencephalic nuclei are segregated, pairs of different retrograde tracers were placed in the two diencephalic sites. It is already known that nucleus reuniens receives dense inputs from the entire inner wall of the rat frontal cortices, starting from the secondary motor cortex (most dorsal) to the dorsal peduncular cortex (most ventral) (Herkenham, 1978; McKenna & Vertes, 2004; Vertes, 2002, 2004). The majority of these frontal inputs have been reported to arise from deep layers (deep V and VI), with some additional inputs to reuniens originating in more superficial layer V (McKenna & Vertes, 2004). Corresponding frontal inputs to the mammillary body region appear to be more concentrated in ventral frontal areas (Allen & Hopkins, 1989; Hayakawa, Ito, & Zyo, 1993; Hurley et al., 1991; Shibata, 1989; Takagishi & Chiba, 1991; Vertes, 2004). As already noted, both diencephalic sites also receive dense inputs from the hippocampus, with the subiculum projecting to both sites. These subiculum projections arise from along the anterior‐posterior length of the subiculum and across its proximal – distal plane to terminate in both diencephalic nuclei (Canteras & Swanson, 1992; Christiansen et al., 2016; McKenna & Vertes, 2004; Shibata, 1989; Varela, Kumar, Yang, & Wilson, 2014; Witter, Ostendorf, & Groenewegen, 1990). Additional areas, including parahippocampal and caudal cingulate (retrosplenial) regions, may also project to both diencephalic sites, and so were also examined.

The present anatomical experiments also allowed us to re‐examine the apparent uncertainty over the extent of the frontal cortex projections to the rodent mammillary bodies. Most studies emphasize the contribution from the dorsal peduncular cortex (Allen & Hopkins, 1989; Shibata, 1989; Takagishi & Chiba, 1991) or, using another nomenclature, the ventral portion of the infralimbic cortex (Hayakawa et al., 1993; Hurley et al., 1991) (see Materials and Methods). However, while some studies also describe a light input from the anterior cingulate cortex (Allen & Hopkins, 1989), this projection is not always seen (Hayakawa et al., 1993). Likewise, it is uncertain whether the mammillary bodies receive a weak projection from prelimbic cortex (Allen & Hopkins, 1989; Sesack, Deutch, Roth, & Bunney, 1989; Shibata, 1989) or not (Vertes, 2004). One technical consideration is that the supramammillary nucleus, which is immediately adjacent to the mammillary bodies, receives appreciable inputs from both the infralimbic and prelimbic cortices (Hurley et al., 1991; Sesack et al., 1989; Takagishi & Chiba, 1991; Vertes, 2004). A related issue is whether the subiculum targets the supramammillary nucleus as well as the mammillary bodies (Allen & Hopkins, 1989; Canteras & Swanson, 1992; Kishi et al., 2000; Witter et al., 1990). For these reasons, the present study used both anterograde and retrograde tracers to distinguish inputs to the mammillary bodies from those to adjacent nuclei.

## 2 MATERIALS AND METHODS

The study involved a total of 21 adult, male Lister Hooded rats (weight 290–320 g, from Harlan/Envigo, UK). All procedures were approved by the ethics committee of Cardiff University and adhered to the UK Animals Act 1986 (Scientific Procedures). Prior to surgery, all animals were housed in groups with sufficient food so that their weight was no less than 85% of their free‐feeding weight. Post‐surgery, all animals were housed in groups with food and water available ad libitum.

### 2.1 Nomenclature and anatomical borders

Nucleus reuniens is a distinct thalamic nucleus that is most extensive in the anterior‐posterior dimension. The ventral margin of nucleus reuniens borders against the dorsal hypothalamus or the so‐called xiphoid (Xi) and paraxiphoid (PaXi) thalamic nuclei (Paxinos & Watson, 2004). Except for its most rostral and most caudal levels, where nucleus reuniens borders the interanteromedial (IAM), anteromedial (AM), and central medial (CM) nuclei, the dorsal border of nucleus reuniens is adjacent to the rhomboid nucleus (Rh). The mammillary bodies, which are positioned ventral to the third ventricle, have the supramammillary nucleus (SUM) at their dorsal border and the dorsal premammillary nucleus at their anterior border (Allen & Hopkins, 1988). The principal components of the mammillary bodies are the medial mammillary nucleus and the lateral mammillary nucleus (LM), the LM containing appreciably larger cells (Allen & Hopkins, 1988). The medial mammillary nucleus is further subdivided, containing lateral (ML), medial (MM) and median (Me) subnuclei (Allen & Hopkins, 1988).

For most other regions, the nomenclature and regional borders follow Paxinos and Watson (Paxinos & Watson, 2004), which also provides a template for plotting the tracer injection sites. On the template the xiphoid thalamic nucleus (Xi, see Paxinos & Watson, 2004) is omitted as we were unable to confirm reliably the position of this small nucleus.

A further change concerns the most ventral portion of the rat medial frontal cortex. This area, which borders the dorsal tenia tecta (TT), is either identified as the dorsal peduncular cortex (DP) (Hurley et al., 1991; Paxinos & Watson, 2004) or, alternatively, is included as part of the infralimbic cortex (IL) (Krettek & Price, 1977). This ventral area is frequently described as having an almost completely unlaminated cell layer, in contrast with the dorsal infralimbic cortex, which although poorly laminated compared to the prelimbic cortex, clearly has a laminar organization. As the peduncular portion of the cortex displays cytoarchitectonic features that are distinct from the infralimbic cortex, the dorsal peduncular cortex is distinguished in our nomenclature. Furthermore, as the infralimbic and the dorsal peduncular cortex have been reported to project differentially to the supramammillary nucleus and the mammillary bodies (Hayakawa et al., 1993; Hurley et al., 1991; Vertes, 2004), a distinction between these areas seems especially pertinent. We do, however, find that a clear separation can be seen between two cell layers in the dorsal peduncular cortex, and we therefore refer to the dorsal peduncular cortex in terms of “superficial” and “deep” cellular layers.

A related issue concerns the importance of differentiating the dorsal tenia tecta from the indusium griseum (IG). At its frontal limit, the tenia tecta segregates into a ventral and a dorsal portion, with the dorsal portion bordering the medial prefrontal cortex. This dorsal portion extends caudal to the genu of the corpus callosum where another structure appears, the indusium griseum. The indusium griseum flanks the corpus callosum and is, in contrast with the tenia tecta, related to the hippocampal formation by morphological and hodological criteria (Adamek, Shipley, & Sanders, 1984; Wyss & Sripanidkulchai, 1983). The portion ventral to the corpus callosum (occasionally termed the “anterior hippocampal continuation”) is limited in its anterior extent whereas the small dorsal portion extends extensively along the anterior‐posterior axis of the corpus callosum, where it comprises a small band of cells ventral to the anterior cingulate and retrosplenial cortices.

### 2.2 Retrograde tracer injections in nucleus reuniens and the mammillary bodies

A total of ten rats received retrograde tracer injections. In seven of these cases, two retrograde tracer injections were made, one directed at nucleus reuniens, the other at the mammillary bodies (Table 1). In the three remaining cases only the injection directed at nucleus reuniens was successful. Different tracers were used, one in each site. The tracers used were fast blue (FB, Sigma‐Aldrich, Gillingham, UK) and cholera‐toxin b (CTB, List Biological Laboratories Inc, CA; 1% solution in 0.05 M tris). Except for one CTB injection that was injected by iontophoresis, all retrograde tracers were injected mechanically at a rate of 20 nl/min via a 0.5 μl or 1.0 μl Hamilton pipette (Hamilton, Bonaduz, Switzerland). Individual injection volumes for mechanical injections were 50–60 nl.

**Table 1:** Overview of retrograde cell label in the seven cases with combined retrograde tracer injections in nucleus reuniens and the mammillary bodies

Case #	Tracer position	dSUB	vSUB	Deep SUB layer	Superficial SUB layer	DP	IL	PL	Cg	M2
208#9	MB	**+**	**+**	**−**	**+**	**+**	**(+)**	**(+)**	**(+)**	**−**
	RE	**+**	**+**	**+**	**−**	**+**	**+**	**+**	**+**	**+**
209#3	MB	**+**	**+**	**−**	**+**	**(+)**	**+**	**+**	**(+)**	**−**
	RE	**+**	**−**	**+**	**−**	**−**	**−**	**+**	**+**	**+**
207#2	MB*	**+**	**+**	**−**	**+**	**+**	**+**	**(+)**	**−**	**−**
	RE*	**+**	**+**	**+**	**(+)**	**+**	**+**	**+**	**+**	**+**
207#4	MB*	**+**	**+**	**(+)**	**+**	**+**	**+**	**(+)**	**(+)**	**−**
	RE*	**+**	**+**	**+**	**(+)**	**+**	**+**	**+**	**+**	**+**
207#9	MB*	**+**	**+**	**−**	**+**	**+**	**+**	**+**	**+**	**−**
	RE*	**+**	**−**	**+**	**−**	**−**	**−**	**−**	**−**	**−**
207#7	MB*	**+**	**+**	**−**	**+**	**+**	**(+)**	**(+)**	**(+)**	**−**
	RE*	**+**	**+**	**+**	**(+)**	**+**	**+**	**+**	**+**	**(+)**
209#10	MB	**+**	**+**	**−**	**+**	**(+)**	**+**	**(+)**	**−**	**−**
	RE*	**+**	**+**	**+**	**(+)**	**+**	**+**	**+**	**+**	**+**

Notes

**Figure 1:**
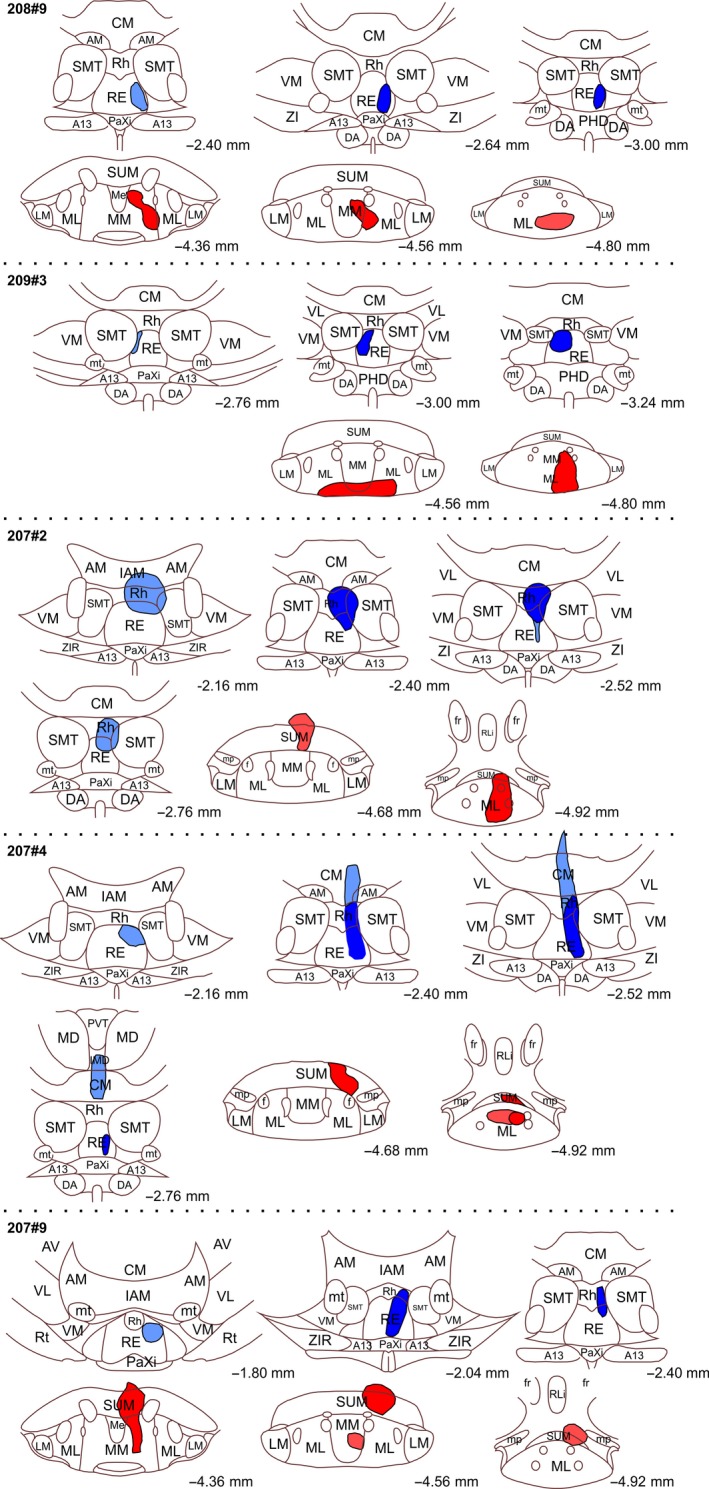
Line drawings of five of the cases with retrograde tracer injections. CTB injections are shown in red and FB injections in blue. In all cases the extent of the visible tracer deposits are depicted. “Dark” colours indicate portions with dense tracer uptake whereas “light” colours indicate a weaker tracer signal. The injections are plotted onto modified templates from Paxinos and Watson (2004) (see text) and the numbers indicate the approximate position from bregma. A11, A11 dopamine cells; A13, A13 dopamine cells; AM, anteromedial thalamic nucleus; AV, anteroventral thalamic nucleus; CM, central medial thalamic nucleus; DA, dorsal hypothalamic areas; DMC; dorsomedial hypothalamic nucleus compact part; DMD dorsomedial hypothalamic nucleus dorsal part; DMV, dorsomedial hypothalamic nucleus ventral part; fr, fasciculus retroflexus; IAM, interanteromedial thalamic nucleus; IMD, intermediodorsal thalamic nucleus; LM, lateral mammillary nucleus; Me, median portion of the medial mammillary nucleus; ML, lateral portion of the medial mammillary nucleus, MM, medial portion of the medial mammillary nucleus; mp, mammillary peduncle; mt, mammillothalamic tract; PaXi; paraxiphoid thalamic nucleus; PH, posterior hypothalamic nucleus; PHD, posterior hypothalamic areas dorsal part; PVT, paraventricular thalamic nucleus; RE, nucleus reuniens; Rh, rhomboid nucleus; RLi, rostral linear nucleus of the raphe; Rt, reticular thalamic nucleus; SMT, submedius thalamic nucleus; SUM, supramammillary nucleus; VL, ventrolateral thalamic nucleus; VM, ventromedial thalamic nucleus; VPM, ventral posteromedial thalamic nucleus; VPPC, ventral posterior nucleus of the thalamus parvicellular part; ZI, zona incerta; ZIR, zona incerta rostral part. [Colour figure can be viewed at wileyonlinelibrary.com]

**Figure 2:**
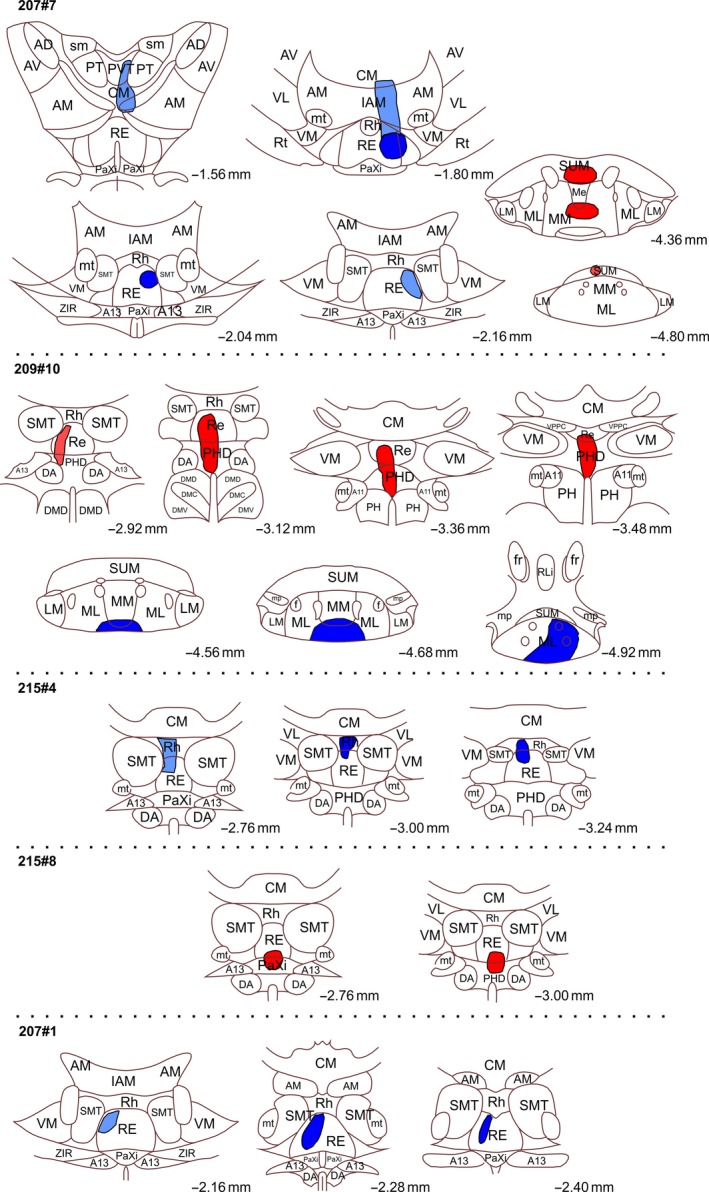
Line drawings of five of the cases with retrograde tracer injections. CTB injections are shown in red and FB injections in blue. In all cases the extent of the visible tracer deposits are depicted. “Dark” colours indicate portions with dense tracer uptake whereas “light” colours indicate a weaker tracer signal. The injections are plotted onto modified templates from Paxinos and Watson (2004) (see text) and the numbers indicate the approximate position from bregma. Abbreviations as Figure 1. [Colour figure can be viewed at wileyonlinelibrary.com]

“+” indicates cell label, “(+)” indicates infrequent label, and “–” indicates either no label or extremely sparse label. The asterisk sign indicates additional involvement of the injection site, see Figures 1 and 2 for details. Data are shown for the subiculum and medial frontal cortices only.

Cg, anterior cingulate cortex; DP, dorsal peduncular cortex; dSUB, dorsal subiculum; IL, infralimbic cortex; M2, secondary motor cortex; MB, mammillary bodies; PL, prelimbic cortex; RE, nucleus reuniens; SUB, subiculum; vSUB, ventral subiculum.

All surgeries took place under isoflurane anaesthesia (isoflurane‐oxygen mixture 1.5%–2.5%) with the rat positioned in a stereotaxic frame (Kopf Instruments, Tujunga, CA, USA). Stereotaxic coordinates were initially derived from a brain atlas (Paxinos & Watson, 2004) and later refined according to the location of previous tracer deposits. In all cases the craniotomy was made on the right hemisphere, but an oblique syringe path helped to target the midline of the target nuclei (6° for nucleus reuniens and 4° for the mammillary bodies).

### 2.3 Anterograde tracer injections in the anterior cingulate cortex, dorsal peduncular cortex and subiculum

A total of eleven rats received anterograde tracer injections. Individual injection volumes for mechanical BDA injections via a Hamilton syringe varied between 60–100 nl, dependent on the target area. In three cases, injections of biotinylated dextran amine (BDA) targeted the anterior cingulate cortex and in one case an injection targeted the dorsal peduncular cortex. In two of these cases, two injections of 3 kD BDA (Life Technologies Ltd, Paisley, UK; 10% in sterile distilled water) were injected mechanically via a 1.0 μl Hamilton pipette (20 nl/min) into the anterior cingulate cortex. In two cases, 10 kD BDA (Invitrogen, UK) was injected using iontophoresis into either the dorsal peduncular or the anterior cingulate cortices. For these iontophoretic cases the tracer was injected via a glass pipette (18–22 μm tip diameter) using an alternating current (6 s on/off). The injection time was 15 min using a current that varied between 2 μA, 6 μA and 7 μA (5 min for each of the settings). In two animals, iontophoretic injections of phaseolus vulgaris‐leukoagglutinin (PHA‐L) targeted the infralimbic cortex while, in the same animals, mechanical injections of 10 kD BDA (Invitrogen, UK) targeted the prelimbic cortex. In these PHA‐L cases, the settings for the iontophoretic injections were the same for the BDA injections, except that the injection time was 18 min total (6 min for each of the three settings).

In three further cases, either 3 kD BDA injections (two cases) (Life Technologies Ltd, Paisley, UK; 10% in sterile distilled water) or WGA‐HRP injections (40 mg/ml; Vector Labs, Peterborough, UK) targeted the dorsal subiculum. In all three cases the tracer was mechanically delivered via a 1.0 μl Hamilton pipette (see Mathiasen, Dillingham, Kinnavane, Powell, & Aggleton, 2017).

Finally, in two cases, we injected a viral vector (AAV5‐CaMKIIa‐EGFP, Addgene, Cambridge MA, USA) into the dorsal subiculum. The virus was mechanically delivered via a Hamilton syringe and we injected 0.6 μl into each hemisphere. The AAV5 virus is transported anterogradely (Chamberlin, Du, de Lacalle, & Saper, 1998) and its location is identified by its GFP tag.

### 2.4 Histology and data analysis

After a 6–10 days survival time (8 weeks for the two animals with viral injections), animals received a 1.5–2.0 ml intraperitoneal pentobarbital injection (Euthatal, Merial, Harlow, UK) and were transcardially perfused with an 0.1 M phosphate‐buffered saline (PBS) solution, immediately followed by PFA perfusion (4% paraformaldehyde solution in 0.1 M PBS). The brains were post‐fixed for four hours in the same PFA solution, then stored overnight in a 25% sucrose solution (25% in 0.1 M PBS). Sections were cut in the coronal plane with a freezing microtome (40 or 50 μm, four series). Two series were initially used. One series was directly mounted on gelatin‐subbed slides (for Nissl stain) and another was placed in PBS at 4°C (for antibody immunohistochemistry).

The mounted sections were dried overnight, rehydrated in a series of ethanol solutions of decreasing concentrations (2 × 100%, 90%, 70%), then stained with cresyl violet after two minutes in deionized water. Following cresyl violet staining, sections were again placed in deionized water, dehydrated (70%, 90%, 2 × 100% ethanol series), defatted in xylene, and finally coverslipped with DPX (ThermoFisher, Waltham, MA, USA).

Except for FB and the viral vector, all the tracers required immunofluorescence or immunohistochemical processing to be visualized. For CTB and PHA‐L staining, sections were washed for 3 × 10 min in 0.1 M PBS, washed 3 × 10 min in PBS‐TX (0.2% Triton X‐100 in 0.1 M PBS) and incubated with either the primary antibody rabbit anti‐cholera toxin overnight (1:3000) (Sigma‐Aldrich, UK) or rabbit anti PHA‐L (1:1000) (Vector laboratories, UK). Sections were washed for 3 × 10 min in PBS‐TX and incubated with the DyLight 594 conjugated secondary antibody goat anti‐rabbit (1:200) (Vector Laboratories, CA, USA) for two hours. For fluorescence BDA staining, sections were washed for 3 × 10 min in 0.1 M PBS, then washed for 3 × 10 min in PBS‐TX (0.2% Triton X‐100 in 0.1 M PBS) and incubated with A488 conjugated streptavidin (Thermofisher, UK) for two hours at room temperature. For both BDA and CTB cases, after a 3 × 10 min wash in PBS, sections were mounted on gelatin‐subbed slides and dried overnight. Sections were then further dehydrated in ethanol (50%, 70%, 90%, 2 × 100%), defatted in xylene and coverslipped with DPX.

The three injections of BDA and WGA‐HRP that targeted the subiculum were visualized with the DAB and TMB methods for brightfield microscopy. The processing of these three cases (as well as the three BDA injection into the cingulate cortex) has been previously described (Mathiasen et al., 2017).

A Leica DM5000B microscope with a Leica DFC310FX digital camera and Leica Application Suite image acquisition software was used for both brightfield and fluorescence microscopy. The latter involved the Leica fluorescence filter DAPI or A (for FB label) and N21 (for CTB and PHA‐L label). In selected cases, the distribution of retrograde labelled cells was plotted with the aid of the CorelDRAW software. Also, the locations of selected tracer deposits were mapped onto line drawings of relevant portions of diencephalon (Figures 1 and 2), closely based on Paxinos and Watson (2004). Fluorescence photomicrographs were acquired for illustration purposes and occasionally adjusted for contrast, brightness and intensity (specified in the figure legends). In all cases that received combined retrograde tracer injections we counted the numbers of retrogradely labelled cells in the subiculum using the Olympus cellSens software. The percentages of double‐labelled cells were calculated in relation to the total number of cells resulting from the respective mammillary body injection.

## 3 RESULTS

A potential difficulty in determining the precise source of those neurons that innervate the mammillary bodies comes from the need to place retrograde tracers within the structure, while stopping their spread into the adjacent supramammillary nucleus, which receives projections from both the infralimbic and prelimbic cortices (Hurley et al., 1991; Sesack et al., 1989; Takagishi & Chiba, 1991; Vertes, 2004). Likewise, thalamic nuclei adjacent to reuniens also receive frontal inputs (Takagishi & Chiba, 1991; Vertes, 2004). For these reasons, the Results sections distinguish those cases with injections most confined to the target sites from those with increasing tracer involvement in adjacent nuclei (Figures 1 and 2). The latter cases not only help to confirm common results but also reveal any particular changes associated with less restricted injections. Further information comes from additional cases with anterograde tracer injections in frontal cortical areas or the subiculum.

**Figure 3:**
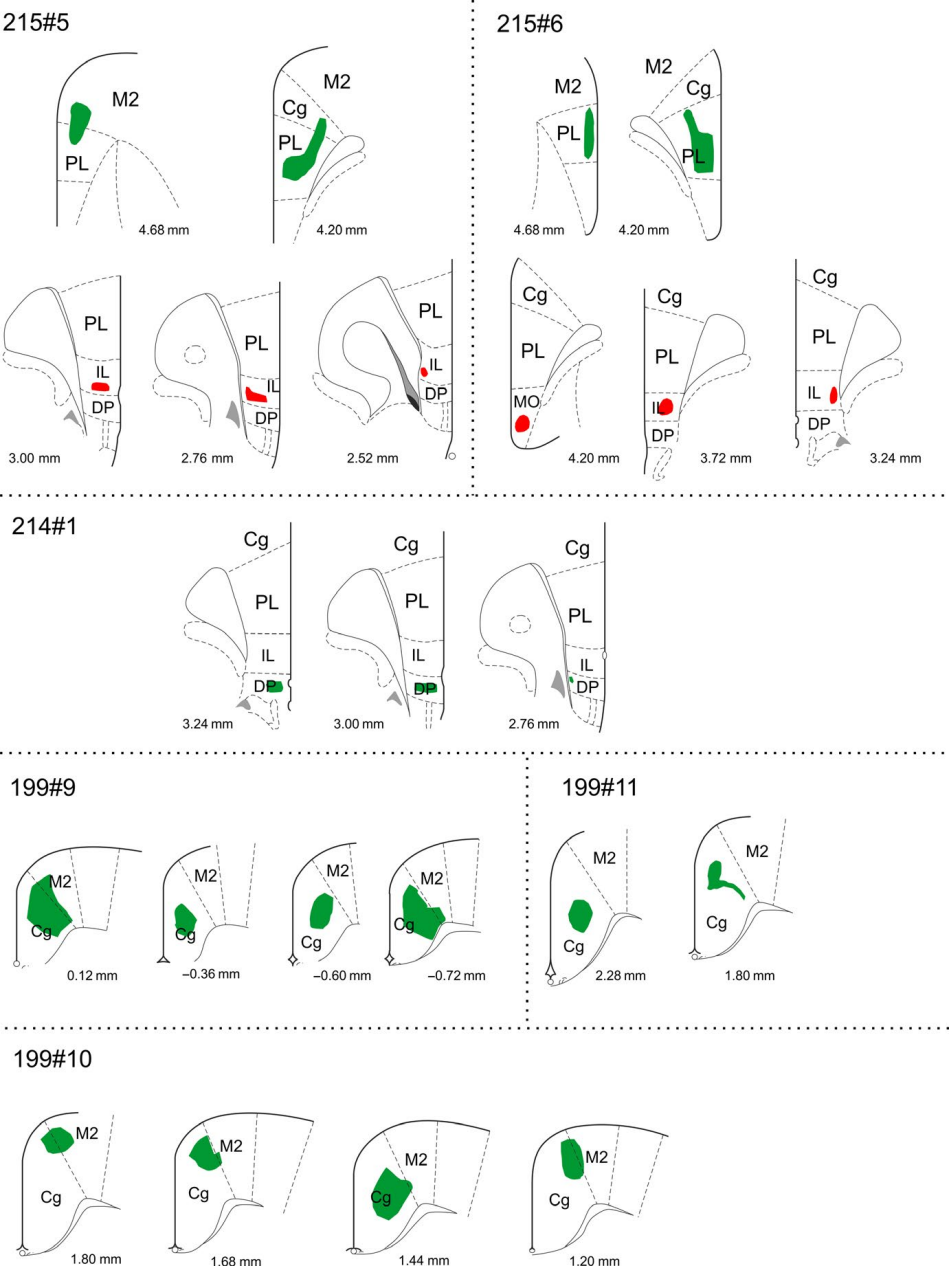
Line drawings of the BDA and PHA‐L injections in frontal cortical areas. PHA‐L injections are shown in red and BDA injections in green. The injections are plotted onto modified templates from Paxinos and Watson (2004) and the numbers indicate the approximate position from bregma according to Paxinos and Watson (2004). Cg, anterior cingulate cortex; DP, dorsal peduncular cortex; IL, infralimbic cortex; PL, prelimbic cortex; M2, secondary motor cortex; MO, medial orbital cortex. [Colour figure can be viewed at wileyonlinelibrary.com]

The full cohort consisted of 21 rats with a total of 30 tracer injections. The subset of seven cases (14 injections) with injections of FB and CTB centred in the two target sites is highlighted (Table 1). The first section describes the patterns of subiculum cell label while the corresponding findings for frontal and other cortical areas are presented in later sections. In each section, we include the results of those anterograde tracer injections (Figure 3) that inform the initial conclusions based on retrograde tracing. Finally, we describe a few single retrograde injections in nucleus reuniens, which assist the interpretation of our results.

### 3.1 Subicular afferents to nucleus reuniens and the mammillary body

#### 3.1.1 Tracer injections restricted to nucleus reuniens and the mammillary bodies

**Figure 4:**
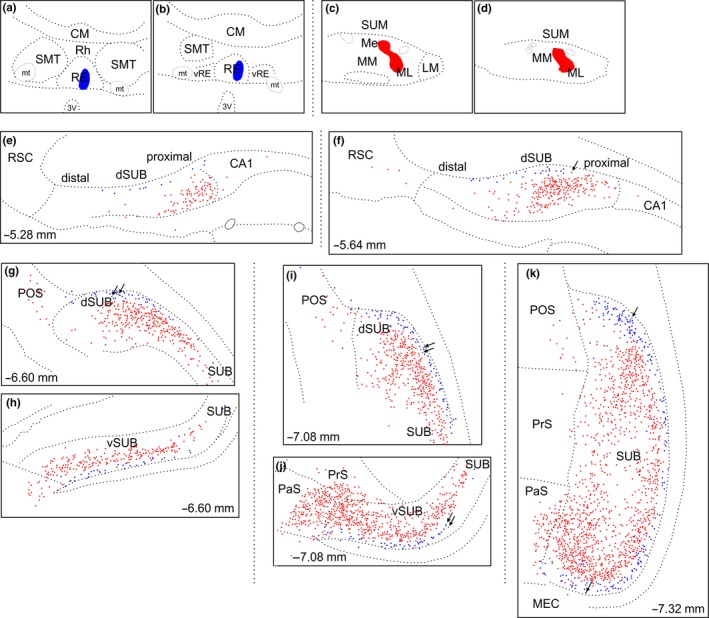
Line drawings of coronal sections showing the distribution of retrogradely labelled cells in the subiculum in case 208#9. a, b: Location of the FB tracer deposit in nucleus reuniens. c, d: Location of the CTB tracer deposit in the mammillary bodies. e–k: Distribution of retrograde labelled cells in the dorsal (e, f, g, i, k) and ventral (h, j, k) subiculum and surrounding areas. The numbers indicate the approximate position from bregma according to Paxinos and Watson (2004). In all sections, the two cell populations display a laminar organization with the FB labelled cells (blue, reuniens injection) positioned deep to the CTB labelled cells (red, mammillary body injection). Green dots (arrowed) designate an occasional double‐labelled cell. CA, cornu ammonis; CM, central medial thalamic nucleus; dSUB, dorsal subiculum; LM, lateral mammillary nucleus; Me, median portion of the medial mammillary nucleus; ML, lateral portion of the medial mammillary nucleus; MM, medial portion of the medial mammillary nucleus; mt, mammillothalamic tract; PaS, parasubiculum; POS, postsubiculum; PrS, presubiculum; RE, nucleus reuniens; Rh, rhomboid nucleus; RSC, retrosplenial cortex; SMT, submedius thalamic nucleus; SUB, subiculum; SUM, supramammillary nucleus; vRE, ventral nucleus reuniens; vSUB, ventral subiculum. [Colour figure can be viewed at wileyonlinelibrary.com]

**Figure 5:**
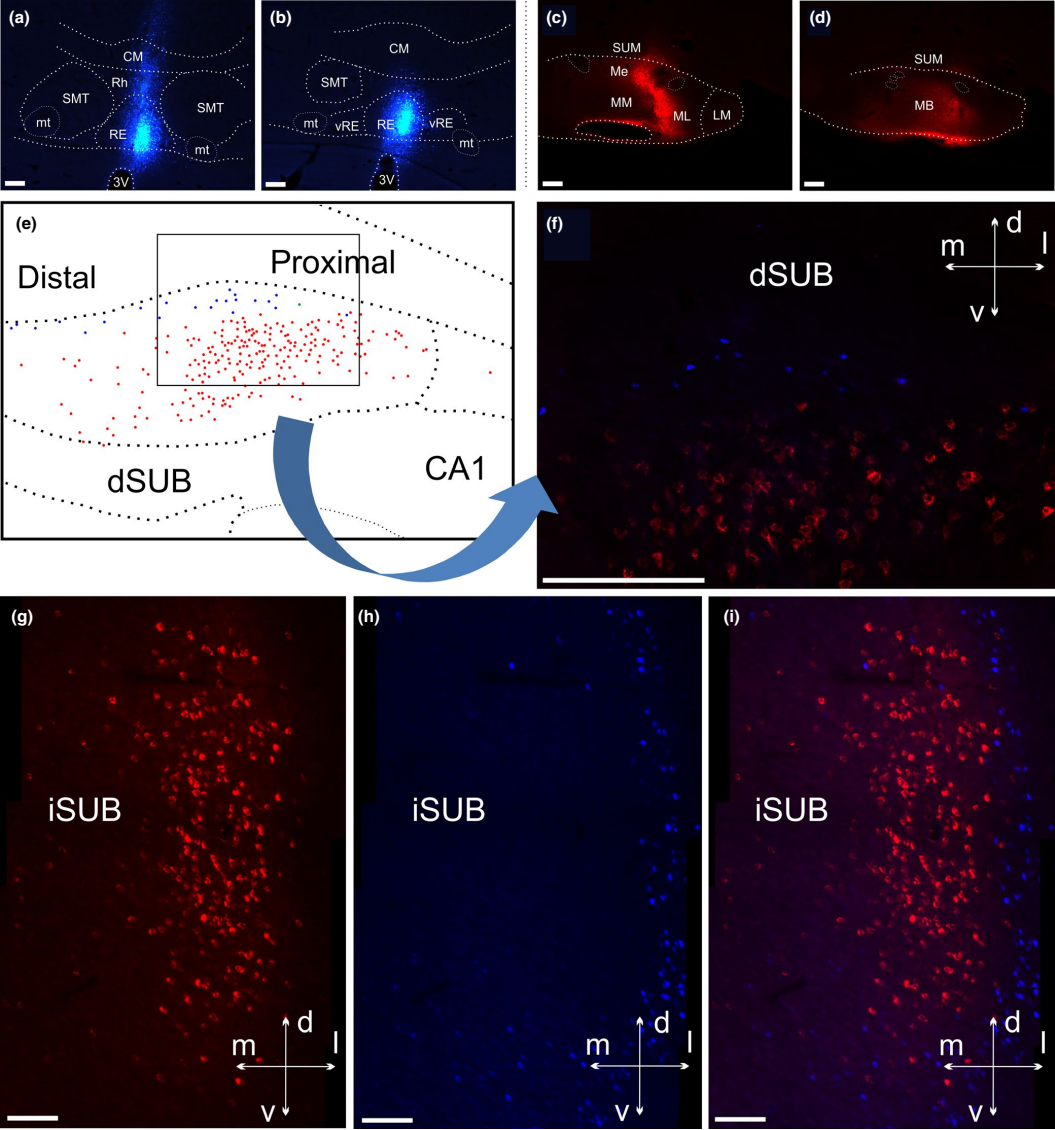
Distribution of retrogradely labelled cells in the subiculum in case 208#9. a, b: Location of FB injection in nucleus reuniens. c, d: Location of CTB injection in the medial mammillary bodies. e: Line drawing (plot from Figure 4), the black box correspond to the image shown in f. f: Fluorescence photomicrograph (20×) that shows an overlay of the CTB labelled cells in the N21 channel and the FB labelled cells in the DAPI channel. g, h, i: Fluorescence photomicrographs (20× tiles) with N21 (g) and DAPI (h) filter channels showing the CTB and FB cell populations (respectively) at the most caudal level of the subiculum, along with the image overlay (i). In all sections, the two cell populations display a laminar organization with the FB labelled cells positioned deep to the CTB labelled cells. Images are adjusted for contrast, brightness and intensity. 3V, third ventricle; CM, central medial thalamic nucleus; dSUB, dorsal subiculum; LM, lateral mammillary nucleus; Me, median portion of the medial mammillary nucleus; ML, lateral portion of the medial mammillary nucleus; MM, medial portion of the medial mammillary nucleus; mt, mammillothalamic tract; Rh, rhomboid nucleus; RE, nucleus reuniens; SMT, submedius thalamic nucleus; vRE, ventral nucleus reuniens; SUM, supramammillary nucleus; Scale bars = 200 μm. [Colour figure can be viewed at wileyonlinelibrary.com]

In case 208#9, the CTB injection site appeared confined to the medial mammillary nucleus of the right hemisphere (Figures 1, 4c,d and 5c,d). Likewise, the FB tracer deposit appeared restricted to nucleus reuniens (Figures 1, 4a,b and 5a,b). Both tracer injections resulted in dense cell labelling in both ventral and dorsal portions of the subiculum, although consistently more labelling resulted from the mammillary body injections compared to the reuniens injections (the reuniens cell population was 20.8% that of the total mammillary labelled population). Both cell populations continued along the entire anterior‐posterior extent of the subiculum and so were equally present in both its ventral and dorsal portions. At septal levels, both tracers labelled cells predominantly within the proximal subiculum, with this organization most prominent for the CTB label (Figure 4e,f).

Despite their parallel organization and distribution, the two cell populations were segregated into two distinct lamina (Figures 4e–k and 5f–i). The subiculum labelled cells resulting from the FB injection in nucleus reuniens were, with only extremely few exceptions, located in the deepest portion of the cellular layer. In contrast, the CTB labelled cells from the mammillary body injection were, with a similar consistency, positioned at a more superficial level. In some sections, especially at septal levels, the lamina boundaries of the two cells populations were separated by a zone of non‐labelled cells. Accordingly, these two cell populations were homogeneously organized in the dorso‐ventral and distal‐proximal dimensions, but segregated by their lamina position, with only 0.35% of the population of CTB labelled cells being double‐labelled (see Figure 4).

A further difference between the two groups of labelled cells was the distinctive morphological appearance of some FB labelled cells in the deep lamina, which contrasted with the more superficial CTB cells. This morphological difference was most evident at septal levels. While the CTB labelled cells were consistently large pyramidal shaped neurons with apical dendrites extending towards the molecular layer, the FB labelled cells had a more varying morphology (see next section).

The laminar segregation of the two populations of cells was again seen in another case with tracer injections restricted to nucleus reuniens and the mammillary bodies (Figure 1; 209#3). In this case, the CTB tracer deposit was centred in the ventro‐caudal portion of the medial mammillary nucleus with the FB injection positioned in the dorsal portion of caudal nucleus reuniens. Except for a relative lack of FB label in the ventral subicular portion, the distribution of labelled cells was comparable to that in case 208#9 and no double‐labelled cells were present in any section inspected. In this case, the proportion of FB labelled cells, compared to the numbers of CTB labelled cells, was 4.86%.

#### 3.1.2 Tracer injections involving the rhomboid/nucleus reuniens and mammillary body/supramammillary nucleus

In five cases the tracer injection sites included the mammillary bodies and nucleus reuniens, but there was varying involvement of the supramammillary and rhomboid nuclei, respectively (see Figures 1 and 2). When these cases are added to the previous dataset, the tracer injections cover most of the anterior‐posterior extent of both nucleus reuniens and mammillary bodies, as well as reaching both medial and lateral portions of nucleus reuniens. Importantly, in all of these cases, independent of tracer position, the two tracers always labelled two principally distinct and separate cell populations in the subiculum.

In a case (207#9) where the FB tracer was restricted to nucleus reuniens and the rhomboid nucleus (and the CTB tracer was centred in both the mammillary and supramammillary nuclei), the FB label was, as in the two cases described above, only present in the deepest lamina of the dorsal subiculum. Here, the FB label was completely separated from the more superficial CTB label in the proximal portion of the subiculum.

**Figure 6:**
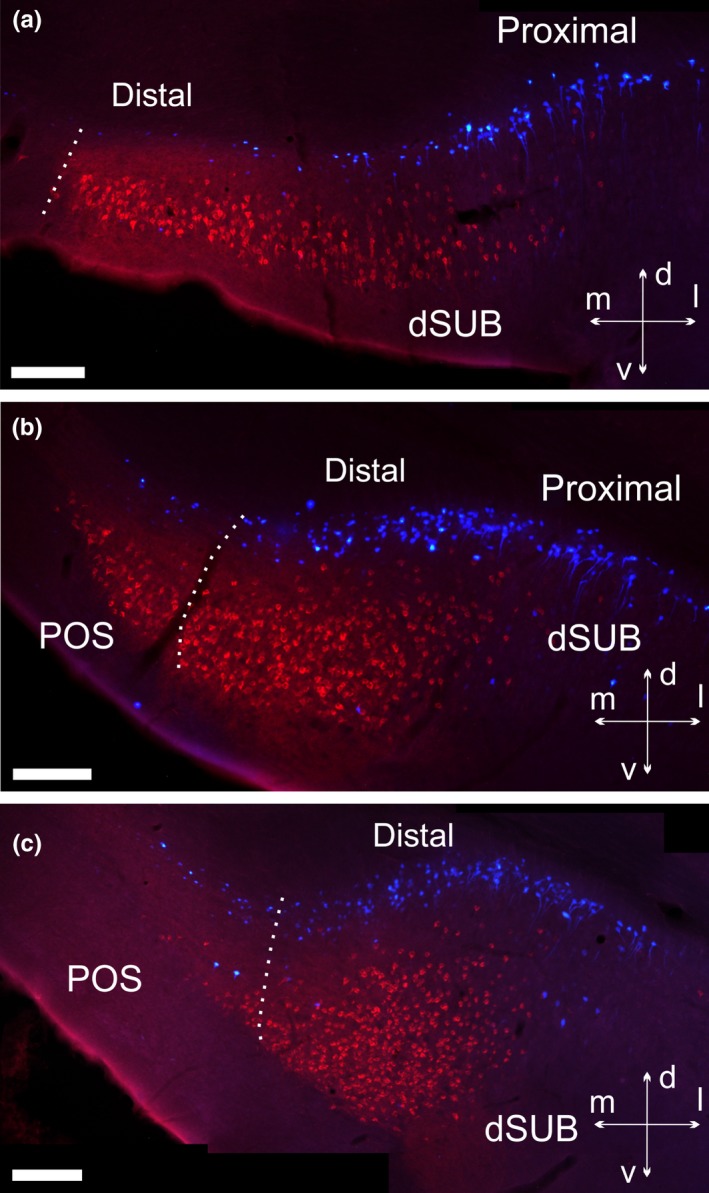
Photomicrographs showing the distribution of retrograde labelled cells in case 207#2. a, b, c: 10× tile images of retrograde labelled cells in the dorsal subiculum at rostral levels. Images are shown as overlays of the two filter channels (N21 and DAPI). The three images are organized from rostral (upper) to caudal (lower). In all sections the two cell populations are distinct and reflect a laminar organization, with the FB labelled cells positioned deep to the CTB labelled cells. The CTB injection site within the mammillary bodies (see Figure 1) includes entering fornix fibres, which may explain the particularly dense subiculum labelling, with additional dense label in the postsubiculum. dSUB, dorsal subiculum; POS, postsubiculum. Scale bars = 200 μm. [Colour figure can be viewed at wileyonlinelibrary.com]

**Figure 7:**
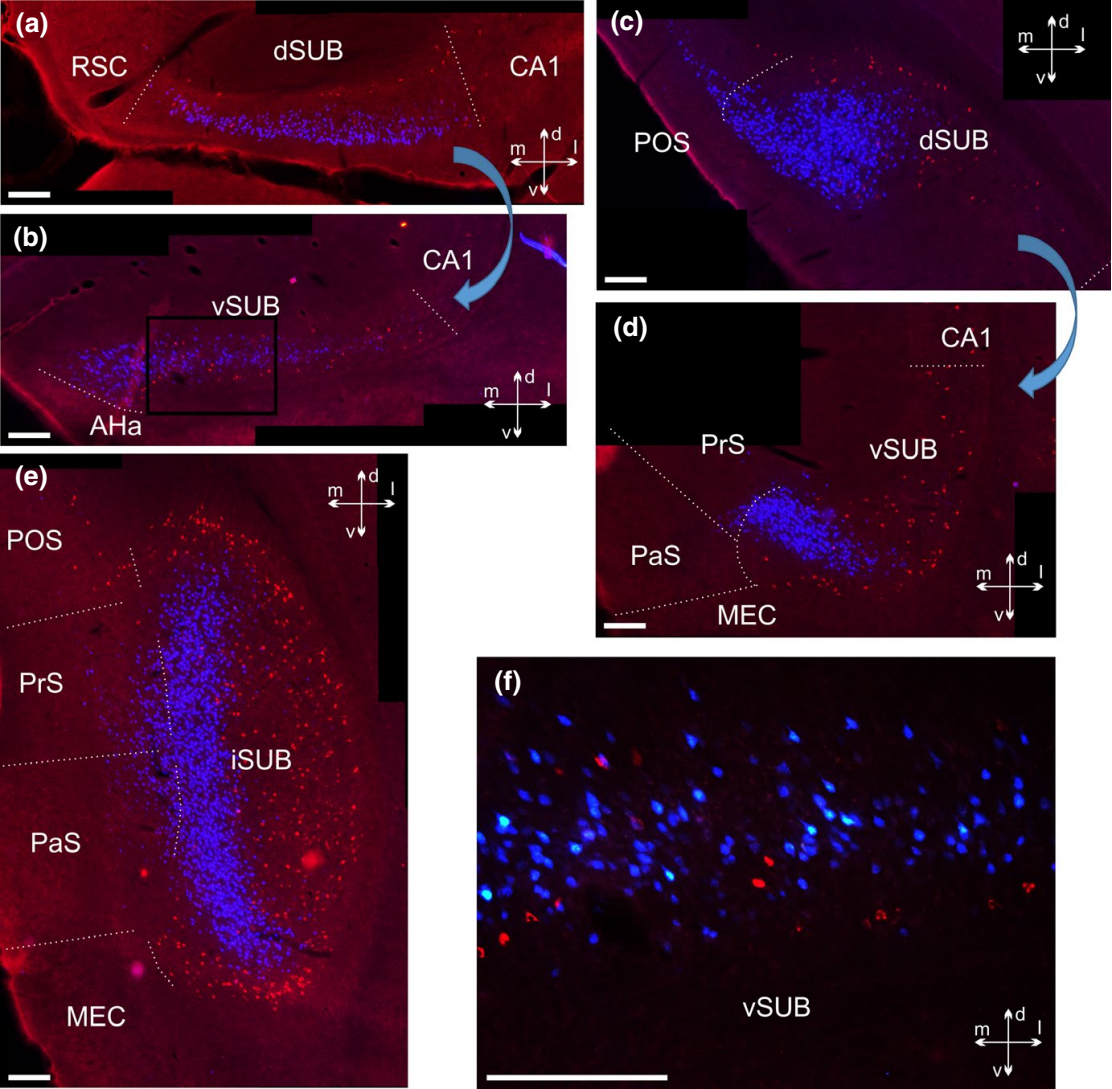
Photomicrographs of hippocampal cell labelling in case 209#10. Unlike other depicted cases, CTB was positioned in nucleus reuniens and FB tracer was in the mammillary bodies (see injection site in Figure 2). The large arrows indicate that two images are from the same section (depicting dorsal and ventral portions of the subiculum) and the sections are organized from anterior (left) to posterior (right) a‐p levels. a, c: Laminar separation between superficial FB and deep CTB labelled cells in the dorsal subiculum. A further population of weaker labelled superficial CTB cells is seen at very proximal levels, but none of these is double‐labelled. b, d: The ventral subiculum likewise displays a clear separation of the two cell populations, although the laminar separation is slightly less obvious at anterior portions (b). e: The separation between the FB and CTB cell populations is preserved at the most posterior sections. Note, the distinct pattern of separation between the two cell populations reflects the curve of the hippocampal formation, such that the deep subicular portions are positioned lateral to superficial portions at mid‐septotemporal levels. f: 20× overlay image of the area of the ventral subiculum indicated by a black box in (b). Except for image f, which is an overlay image of the N21 and “A” filter channels, all images are overlays that combine the N21 and DAPI filter channels. The contrast has been adjusted. AHa, amygdalohippocampal area; CA, cornu ammonis; dSUB, dorsal subiculum, iSUB, intermediate subiculum; MEC, medial entorhinal cortex; PaS, parasubiculum; POS, postsubiculum; PrS, presubiculum; RSC, retrosplenial cortex; vSUB, ventral subiculum. Scale bars = 200 μm. [Colour figure can be viewed at wileyonlinelibrary.com]

**Figure 8:**
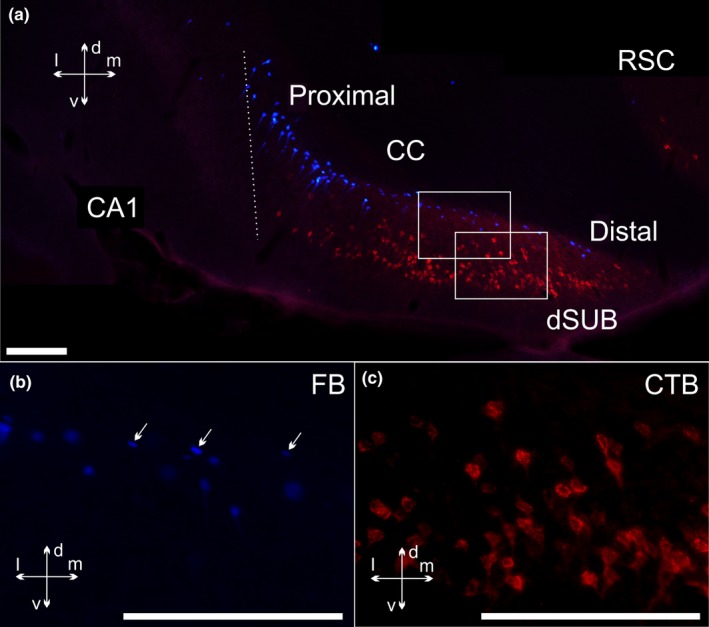
Coronal photomicrographs illustrating the differences in somatic morphology between the deep and superficial dorsal subicular cells. In this case (207#2) a CTB injection covers the mammillary bodies and the supramammillary nucleus while a FB injection is located in both nucleus reuniens and the rhomboid nucleus, with some additional spread (see Figure 1 for details). a: 10× tile image of the overall labelling pattern in the dorsal subiculum. b, c: 40× zoom pictures of the areas of the dorsal subiculum, shown by the two square in a, showing FB (b) and CTB (c) labelled cells. Arrows in b point towards examples of small cells with a non‐pyramidal shape in the deepest layer of the subiculum, distinct from the CTB labelled cells. All FB cells are imaged by the DAPI filter channel and CTB cells by the N21 channel. Image b and c are adjusted for contrast, brightness and intensity. Image c is an overlay picture of two images of the same area, but with different focus. CA, cornu ammonis; CC, corpus callosum; CTB, cholera toxin b; dSUB, dorsal subiculum; FB, fast blue; RSC, retrosplenial cortex. Scale bars = 200 μm. [Colour figure can be viewed at wileyonlinelibrary.com]

Minor variations in the distribution of labelled cells were observed, however, when the tracer deposits included diencephalic nuclei other than the reuniens/rhomboid combination. This variation was seen in two cases where the thalamic tracer injection also included either a portion of the posterior hypothalamic area (209#10) or relatively small portions of neighbouring thalamic nuclei (207#2) (see Figures 1 and 2 for details). In these two cases, we again observed distinct cell label in the deepest lamina (from thalamic injections) that was separate from the more superficial labelling of cells projecting to the mammillary bodies (Figures 6 and 7). We again observed the labelling of a group of deep polymorphic cells in the septal subiculum (from the thalamic injection), distinct from the more homogenous pyramidal cells in a more superficial positions (from the mammillary body injection) (Figure 8). However, in these same two cases (207#2, 209#10), the thalamic injections occasionally labelled cells in superficial positions within the subiculum. These superficial cells were sparse at septal levels but became slightly more numerous at intermediate levels, where they were predominantly scattered in a position external to the plexus of cells projecting to the mammillary region (Figures 6b,c and 7c). Likewise, in the ventral subiculum, occasional cells labelled with the thalamic tracer were scattered superficial to the main cell plexus positioned in the deepest lamina (Figure 7b,f). Although some of these more superficial cells were, therefore, intermingled with the population of cells that projects to the mammillary region, the numbers of double‐labelled cells were extremely limited. In one of these two cases (209#10) the locations of the tracers were exchanged compared to the other six cases, i.e., FB in the mammillary bodies and CTB in nucleus reuniens, but this led to no apparent differences in subicular cell label. In all of these cases with slightly more extensive injections we, again, observed that the cells labelled in the deepest lamina (projecting to the thalamus) displayed a more varying morphology than the more homogenous superficial pyramidal cells (projecting to the mammillary bodies). In none of these cases did the number of double‐labelled cell exceed 0.2% of mammillary labelled cells. Indeed, in both case 207#9 and 207#2 only a single double‐labelled cell was observed in total.

In two further cases (207#4; 207#7), tracer leakage along the syringe tract dorsal to the nucleus reuniens resulted in limited uptake in portions of both the mediodorsal and the anterior thalamic nuclei (see Figures 1 and 2 for details) as well as an increase in the numbers of more superficial cells in the ventral subiculum. These two cases were slightly different, as, apart from the labelling as described above, we observed a modest increase in the number of double‐labelled cells in the subiculum compared to the other cases in this section (2.95% of mammillary labelled cells in case 207#4 and 1.13% in case 207#7).

#### 3.1.3 Anterograde tracer injections in the subiculum

A number of our retrograde tracer injections in the mammillary bodies included portions of the supramammillary nucleus and there is inconsistency in the literature about whether this latter area receives subicular projections (see Introduction1). We, therefore, analysed five anterograde tracer injections centred in the subiculum in order to evaluate the risk that observed retrograde cell labelling in subiculum could originate from uptake in the supramammillary nucleus.

**Figure 9:**
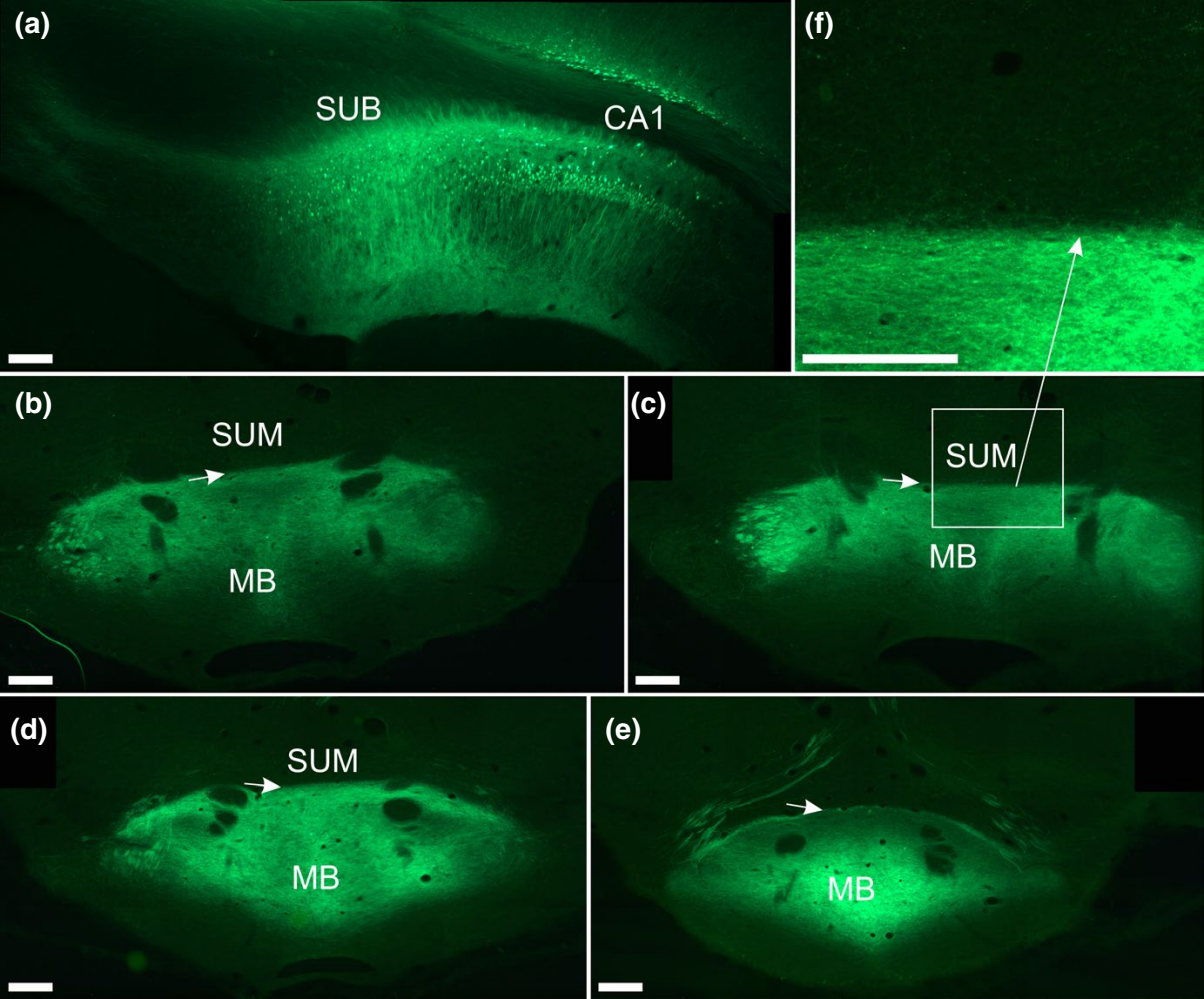
Photomicrographs showing the distribution of terminal fibre labelling in the mammillary bodies following bilateral infusion of AAV5‐CaMKII‐EGFP virus into dorsal subiculum. a: 10× tile scan of the centre of the injection site in the subiculum (right hemisphere). Some virus label is also seen in the dorsal CA1 and neocortical layer VI. b–e: 10× tile scan of anterograde fibre label in the mammillary bodies. The very dense fibre plexus targets the mammillary bodies forming a clear border with the supramammillary nucleus (indicated by small arrows). f: 20× zoom image of the area indicated by a box in image c. Photomicrograph f shows that even though labelling in the supramammillary nucleus is extremely weak, very occasional terminal labelling is present. CA, cornu ammonis; MB, mammillary bodies; SUB, subiculum, SUM, supramammillary nucleus. Scale bars = 200 μm. [Colour figure can be viewed at wileyonlinelibrary.com]

These cases consisted of a large injection of WGA‐HRP placed in the intermediate subiculum (82#2), as well as two cases with BDA injections in the dorsal subiculum (182#3; 182#4). We also analysed two cases with bilateral AAV5‐CaMKIIa‐EGFP injections centred in dorsal subiculum (212#10; 212#11) (Figure 9a). In the three cases with BDA or WGA‐HRP injections, the expected dense fibre innervation was seen in the mammillary bodies (medial mammillary nucleus). In contrast, no fibres were observed in the supramammillary nucleus. A topography was present, such that the two injections in the dorsal subiculum specifically labelled the dorsal portion of the mammillary bodies whereas the large injection in the intermediate subiculum specifically labelled the more ventral mammillary bodies. Likewise, in the two cases with viral injections we observed a particularly dense fibre innervation of the mammillary bodies (Figure 9b–e). In these cases, however, extremely scattered terminal fibre labelling was visible in portions of the supramammillary bodies (Figure 9f). In these two cases anterograde label was densely concentrated in nucleus reuniens, alongside sparser label in the paraxiphoid thalamic nucleus and the hypothalamic portion immediately ventral to nucleus reuniens. Further terminal labelling was present in the submedial and the rhomboid thalamic nuclei, although the rhomboid label was particularly sparse and scattered compared to the fibre plexus in nucleus reuniens.

### 3.2 Frontal and other cortical afferents to nucleus reuniens and the mammillary body

#### 3.2.1 Tracer injections restricted to nucleus reuniens and the mammillary bodies

**Figure 10:**
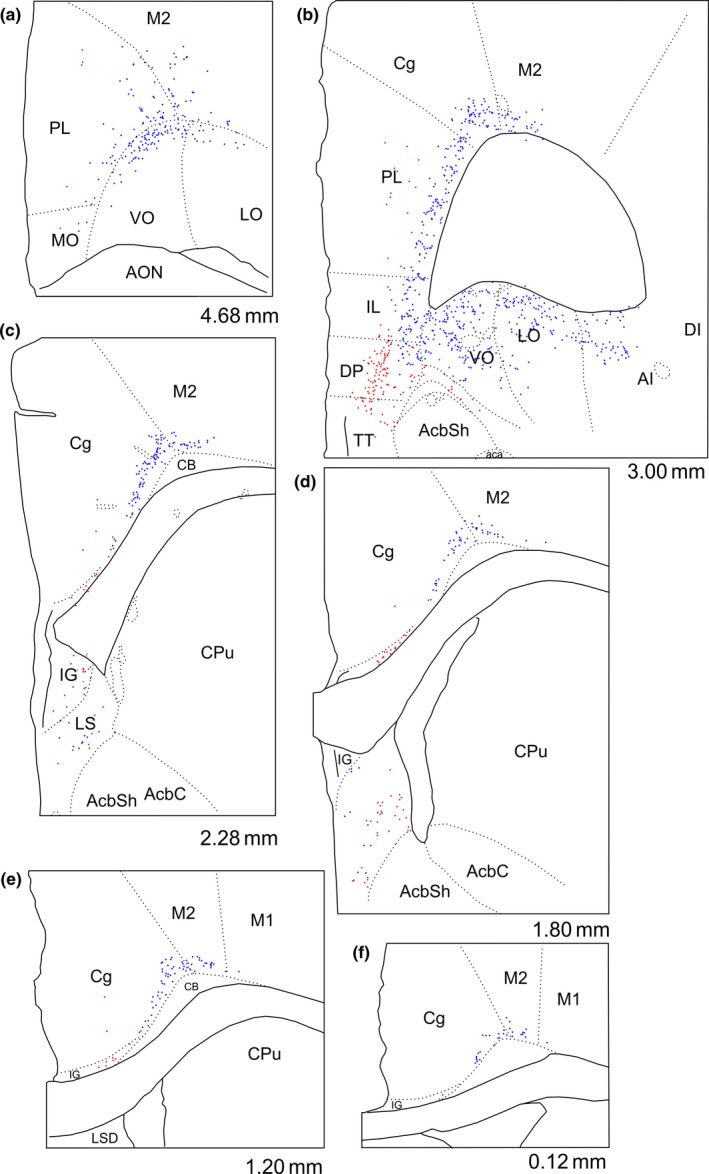
Line drawings depicting the distribution of retrogradely labelled cells in case 208#9 (see Figure 1). Red dots indicate labelled CTB cells (mammillary body injection) and blue dots indicate labelled FB cells (nucleus reuniens injection). The few occasional green dots indicate double‐labelled cells. AcbC, nucleus accumbens core; AcbSh, nucleus accumbens shell; AI, agranular insular cortex; CB, cingulum bundle; Cg, anterior cingulate cortex; Cpu, caudate putamen; DI, dysgranular insular cortex; DP; dorsal peduncular cortex; IG, indusium griseum; LO, lateral orbital cortex; LSS, lateral septal nucleus dorsal part; M1, primary motor cortex; M2, secondary motor cortex; MO, medial orbital cortex; PL, prelimbic cortex; TT, tenia tecta; VO, ventral orbital cortex. [Colour figure can be viewed at wileyonlinelibrary.com]

**Figure 11:**
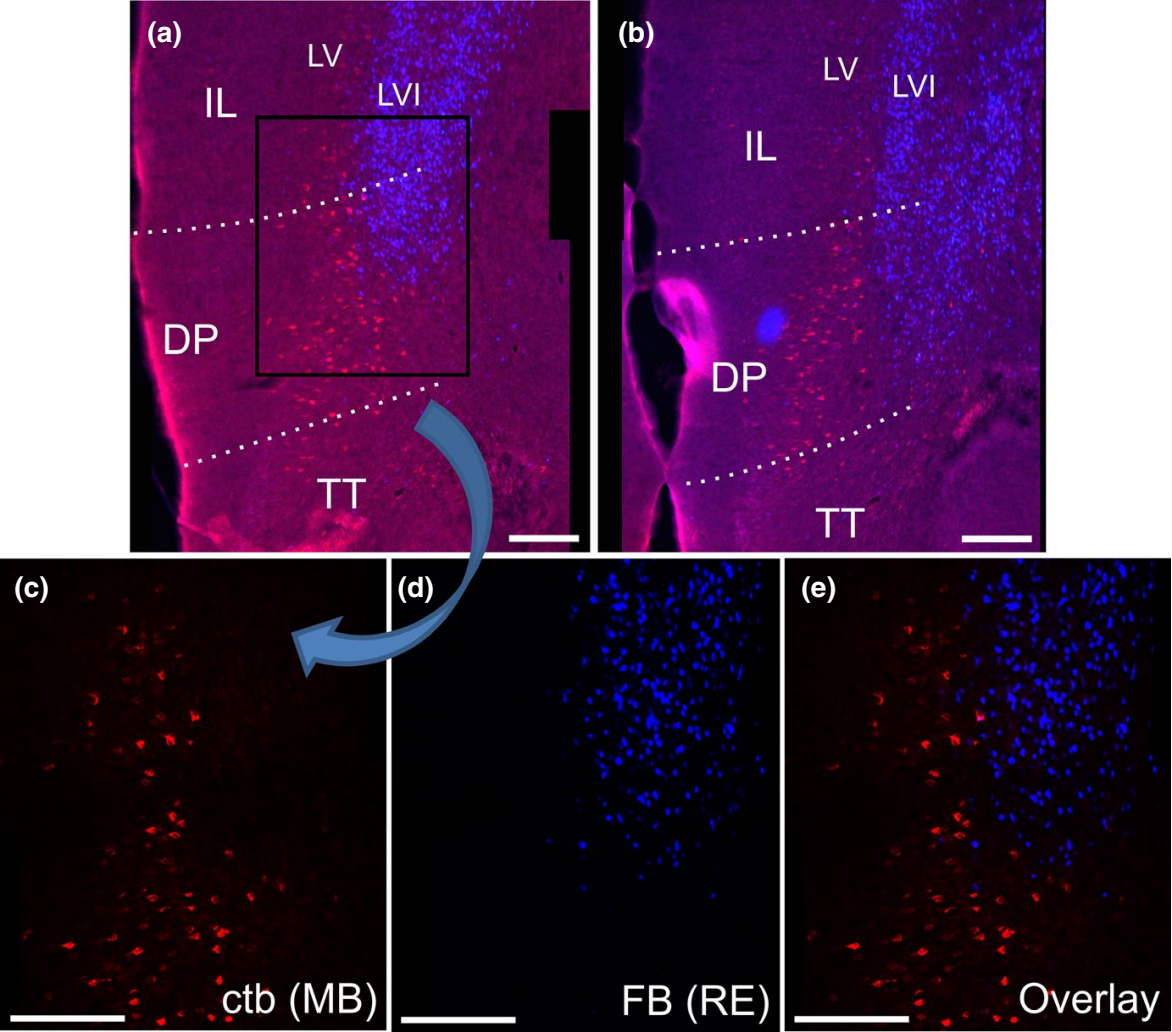
Coronal photomicrographs of frontal cortical cell labelling in case 207#7. The CTB injection is centred in the mammillary bodies and the FB injection in nucleus reuniens (see Figure 2 and Results for details). a, b: A dense plexus of CTB labelled cells is present in the dorsal peduncular cortex (DP) superficial to a deep plexus of FB labelled cells in same area. More moderate CTB labelling is present in layer V of the infralimbic cortex (IL) with occasional CTB labelling in more superficial layers. In the same area, the densest FB labelling is present in layer VI with more moderate FB labelling in layer V, intermixed with the CTB labelled cells in this layer. Dorsal to these areas (not shown), relatively sparse CTB labelling extends into the prelimbic and anterior cingulate cortices, while dense FB labelling is present in all medial frontal areas. These images are overlay pictures that combine both filter channels (N21 and DAPI filters). c, d, e: 20× tile images that show higher resolution views of the area indicated within a box in image A. Images show CTB labelled cells in the N21 channel (c), FB cells in the DAPI channel (d) as well as an overlay of the two (e). All images are adjusted for contrast, brightness and intensity. DP, dorsal peduncular cortex; IL, infralimbic cortex; LV, layer V; LVI, layer VI; TT, tenia tecta. Scale bars = 200 μm. [Colour figure can be viewed at wileyonlinelibrary.com]

In both cases with the tracer deposits seemingly restricted to nucleus reuniens (FB) and the mammillary bodies (CTB) (208#9; 209#3), the two populations of labelled cells were separated in all cortical areas, with virtually no double‐labelled cells present (limited to a single cell in case 208#9) (Figure 10). (See also case 207#7, Figure 11). There were, however, clear differences in the frontal label associated with the two mammillary body injections. These two CTB injections differed in the extent to which the tracer deposit included more dorsal portions. In agreement with our anterograde tracing data (see below), in the case (208#9) where the CTB tracer included the dorsal portion of the rostral mammillary bodies, there was a noticeable concentration of label in the superficial cell layer of the dorsal peduncular cortex (DP), with weaker label in the deep layer (Figure 10b). Dorsal to this cortical area, only sparse cell labelling was present in both deep layers V and VI of the infralimbic (IL) and prelimbic (PL) cortices in case 208#9 (Figure 10a,b). This sparse labelling was also seen at more rostral portions of the ventral prelimbic cortex, although here the cell label, which was most prevalent in layer V, also included some cells in the medial orbital cortex (MO). Occasional scattered label was also seen in the anterior cingulate cortex (Cg).

In the other case (209#3), where the CTB tracer deposits in the mammillary bodies avoided its dorsal portion, we did not see the obvious concentration of labelled cells in the superficial layer of the dorsal peduncular cortex, instead the fewer labelled cells were restricted to the deep layer of this same area. The CTB label dorsal to the dorsal peduncular cortex (predominantly in infralimbic and ventral prelimbic cortex) was most evident in layer VI (relatively more dense in caudal portions), with only sparse label in layer V. Besides this, overall, the frontal CTB label mimicked the previous case 208#9.

The CTB labelling contrasted with the pattern of dense labelling associated with the FB injections in nucleus reuniens (cases 208#9, 209#3). In both cases, at very frontal cortical levels, the FB label was largely confined to layer VI, resulting in a striking laminar separation between CTB labelled cells in layer V and FB labelled cells in layer VI (Figure 10a). This pattern, where the two cell populations are separated by their laminar organization, was also seen at more caudal levels of the frontal cortices in case 208#9, where dense FB label was present ipsilateral in layer VI of all medial frontal cortices (infralimbic, prelimbic, anterior cingulate as well as secondary motor cortex), including the deep layer of the dorsal peduncular cortex. In this same animal, the CTB label was concentrated in the superficial cell layer of the dorsal peduncular cortex. Consequently, a prominent laminar separation was visible between deep positioned FB cells and more superficial CTB cells (Figure 10b). Among the scattered CTB cells that were mixed with the FB labelled cells in layer VI along the dorsoventral extent of the medial frontal cortex, only extremely few were double‐labelled by both tracers (see Figure 10b). Likewise, in case 209#3, the FB cortical labelling (reuniens injection) was concentrated (bilaterally) in layers VI, though in this case it was restricted to the dorsal prelimbic, cingulate and motor regions. Consequently, as the CTB tracer only labelled these dorsal portions extremely sparsely, the two cell populations (reuniens and mammillary bodies) in 209#3 were essentially separated along the dorsoventral axis of the medial frontal cortex. In the retrosplenial cortex (RSC), again, there was no overlap between the FB and the CTB labelled cell populations as FB label was present only at the most rostral portions of the cortical area and the CTB label limited to rather sparse labelling caudally in the granular portion.

In both cases (208#9; 209#3), a conspicuous labelling pattern was seen in the indusium griseum (IG) at the level of the genu of the corpus callosum. At this level, the indusium griseum extends along both dorsal and ventral sides of the corpus callosum. In its ventral portions, relatively dense numbers of CTB labelled cells (mammillary injection) were mixed among a sparser population of FB cells (reuniens injection), although no double‐labelled cells were observed. In the dorsal portion of the indusium griseum, however, the cell labelling was largely restricted to CTB, with only extremely few FB labelled cells. The CTB labelling in the dorsal indusium griseum extended caudally above the corpus callosum (ventral to the anterior cingulate cortex border (Figure 10e,f).

For both cases (208#9; 209#3), very dense FB label from the reuniens injection was present in the deep layers of the lateral (LO) and ventral (VO) orbital as well as agranular (AI) insular cortices (Figure 10a,b). This label contrasted with the CTB label, which in these same areas was limited to very sparse cell label in the agranular insular cortex (AI). The opposite pattern was present in the parahippocampal region, where the CTB injection labelled cells in the entorhinal cortex as well as in the pre‐, para‐ and post‐subiculum, whereas FB label was restricted to extremely scattered label in the perirhinal cortex of case 208#9. In both cases, we only observed sparse FB label in the postsubiculum.

#### 3.2.2 Tracer injections involving the rhomboid/nucleus reuniens and mammillary body/supramammillary nucleus

The five cases with more extensive tracer injections allowed us to test our initial conclusion that the number of double‐labelled cells was extremely limited. One issue was whether the injections had sufficiently filled the target nuclei. Of these five additional cases, one did not show any retrograde transport of FB (reuniens injection) in frontal cortical regions (207#9), but in the remaining four cases we observed labelling in the same cortical portions as in the two cases with more restricted tracer injections. Importantly, we observed additional retrograde labelling (from tracers centred in reuniens), not seen in previous described cases. First, in all four cases the tracer that involved nucleus reuniens labelled cells in layer V, i.e. not only layer VI, of the infralimbic, prelimbic and anterior cingulate cortices. Likewise, this same tracer not only labelled deep layer cells in the dorsal peduncular cortex, but occasionally labelled cells in the superficial cell layer.

Second, these same thalamic tracer injections labelled cells in the indusium griseum (dorsal portion), perirhinal and postrhinal cortices. In addition, denser labelling was present in the retrosplenial cortex (compared to the cases with restricted injections). In two cases (207#4, 207#2; Figures 1 and 2) (especially case 207#4 where the additional tracer uptake was particularly marked), further labelling was present along more lateral cortical areas.

As noted in the previous section, the pattern of frontal labelling after mammillary body injections was dependent on whether the tracer included the dorsal portion of the mammillary bodies, as well as the degree of tracer spread into the supramammillary nucleus. In cases 207#2 and 207#4, the CTB injections included both the supramammillary nucleus and mammillary bodies. In both cases, CTB labelled cells were present with similar density in both the dorsal peduncular and infralimbic cortices, with sparser label in the prelimbic cortex. This CTB label was distributed within deep layers V and VI, and in the dorsal peduncular cortex, principally in the superficial cell layer. This distribution was also seen in case 207#7, but in this case (207#7) there was denser, more restricted dorsal peduncular label (Figure 11) (again principally in the superficial layer). Finally, in case 209#10, where the FB tracer deposits seemed restricted to the ventral portion of the mammillary bodies, frontal FB labelling was centred in the infralimbic cortex (with sparse labelling in the prelimbic cortex), with substantially fewer labelled cells in the dorsal peduncular cortex. The infralimbic label was distributed in both layers V and VI. This case, thereby, mimicked the labelling pattern described in the previous section for case 209#3 that, likewise, had the tracer deposits limited to the ventral portions of the mammillary bodies.

In most cases, the tracer that targeted the mammillary bodies labelled, with varying density, cells in layer V of the anterior cingulate cortex, with some additional cells in layer VI (Table 1). Further labelling at these levels was seen in the indusium griseum. Occasionally a few cells (mammillary body tracer injections) were also seen in the agranular insular cortex and granular retrosplenial cortex. The label in the anterior cingulate cortex was almost non‐existent in the case with a restricted mammillary body injection (209#10). However, as case 207#7, which appeared to have predominantly mammillary bodies tracer uptake (based on its cortical labelling patterns, see above), displayed appreciable layer V label, it remained uncertain whether this cingulate label is of supramammillary or mammillary origin (see section on anterograde tracing to resolve).

Despite these two cases (207#7, 209#10) with more extensive injections resulting in the two cell populations (CTB vs. FB labelled cells) being more intermixed in frontal cortices (dorsal peduncular, infralimbic and prelimbic cortices), anterior cingulate cortex, and the indusium griseum, this greater overlap did not result in anything more than sporadic double‐labelling of cells.

Further label, originating from tracers involving the mammillary bodies, was seen in the medial entorhinal cortex, pre‐ and parasubiculum as well as postsubiculum (all cases, except case 207#7). Tracers that involved nucleus reuniens likewise labelled the postsubiculum, but this label was consistently located in a deeper laminar position than the label originating from mammillary body injections (Figures 6b,c and 7c,e). In contrast, with the exception of case 207#7, which displayed some pre‐ and parasubiculum label, these thalamic injections did not label cells in the medial entorhinal cortex or the pre‐ and parasubiculum.

#### 3.2.3 Anterograde tracer injections in frontal cortices

**Figure 12:**
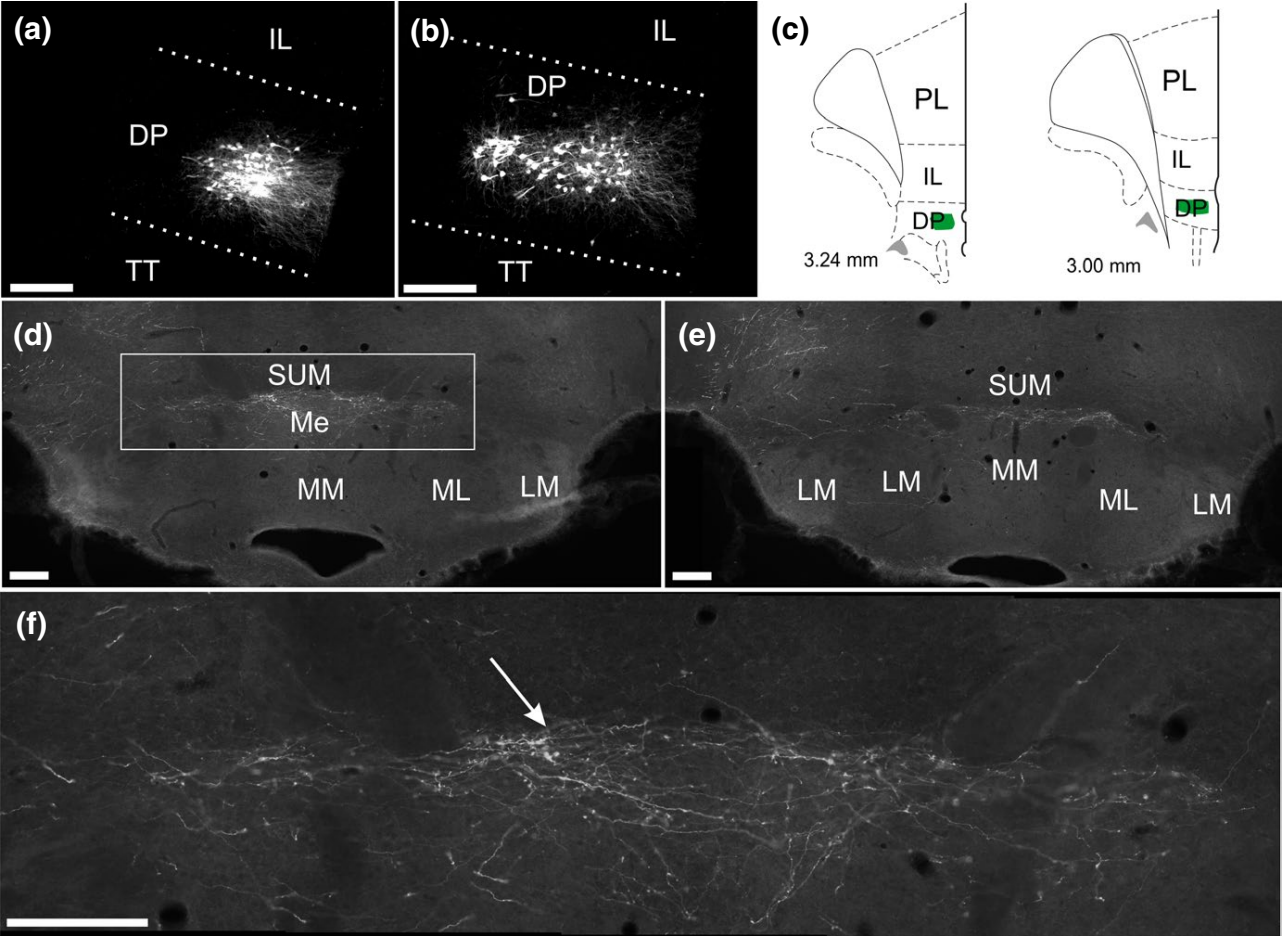
Photomicrographs that show fibre labelling in the mammillary bodies following an anterograde tracer injection (BDA) in the dorsal peduncular cortex (case 214#1). a, b: Photomicrographs of the BDA injection site. c: Line drawings (plot from Figure 3) that correspond to images a and b. d, e: 10× tile images of fibre labelling in the dorsal mammillary bodies. f: 20× tile photomicrograph of the area indicated by a box in d. The arrow points to the border between the mammillary bodies and the supramammillary nucleus. The BDA tracer is visualized by an Alexa488 fluorophore (see Methods) but images are in shown in grey‐scale. The pictures are adjusted for contrast, brightness, intensity and gamma. DP, dorsal peduncular cortex; IL, infralimbic cortex; LM, lateral mammillary nucleus; Me, median portion of the medial mammillary nucleus; ML, lateral portion of the medial mammillary nucleus; MM, medial portion of the medial mammillary nucleus; PL, prelimbic cortex; SUM, supramammillary nucleus; TT, tenia tecta. Scale bars = 200 μm. [Colour figure can be viewed at wileyonlinelibrary.com]

A total of eight anterograde tracer injections were placed in cortical regions (Figure 3). In one case (214#1), the tracer deposit from a single iontophoretic BDA injection (10 kD) was confined to the dorsal peduncular cortex (Figure 12). In this case, dense fibre labelling was present in the medial mammillary nucleus, including its median portion (Figure 12d–f). In contrast, two iontophoretic PHA‐L injections (215#5 PHA‐L; 215#6 PHA‐L) centred slightly more dorsal in the infralimbic cortex resulted in only very scattered fibre labelling in the mammillary bodies. Likewise, two further injections (same animals) with large mechanical infusions of BDA (10kD) into rostral prelimbic cortex resulted in no mammillary fibre label. In one of these cases (215#6 PHA‐L) the PHA‐L injection included a very small portion of MO and in one of the BDA cases (215#5 BDA) the injection included a portion of the rostral M2 and Cg regions.

Finally, in three cases, BDA injections were made in the anterior cingulate cortex at levels caudal to the genu of the corpus callosum (199#9; 199#10; 199#11). In two cases, the tracer deposit spread to a small portion of the neighbouring secondary motor cortex (M2), whereas in the third case it was restricted to the anterior cingulate cortex. Importantly, in none of these cases did the injection site include the indusium griseum. In all three cases, a consistent pattern of weak fibre labelling was present in the mammillary bodies. These fibres were sparsely scattered in the medial mammillary nucleus (median and medial subnuclei) and, compared with the fibre innervation of the more dorsal supramammillary nucleus, the labelling intensity was weak. These fibres were absent from the caudal portion of the mammillary bodies.

In all of these cases with frontal injections, substantial fibre labelling was also present in nucleus reuniens. When we looked at those nuclei surrounding reuniens, a dense plexus was seen in the submedius nucleus following the dorsal peduncular injection (case 214#1). Labelling in the submedius nucleus was markedly less in the cases with infralimbic injections, and even sparser following the cases with prelimbic cortex injections. Fibres in the rhomboid nucleus were evident in some cases with injection sites centred in either the anterior cingulate or prelimbic cortices, but label in the same nucleus was appreciably more scattered after cortical injections positioned ventral to the prelimbic area. Relatively sparse and scattered labelling was also seen in the paraxiphoid nucleus and posterior hypothalamus following injections in all of these frontal areas.

#### 3.2.4 Additional retrograde tracer injections in nucleus reuniens and surrounding nuclei

Two patterns emerged above when comparing confined retrograde tracer injections in nucleus reuniens with injections that were less confined to the same nucleus. First, the former cases labelled exclusively the deepest layer in subiculum whereas less restricted cases, to a varying degree, also labelled superficial subicular cells. Second, these restricted cases labelled layer VI in medial frontal areas, whereas some of the less confined injections also labelled layer V relative densely. In order to shed further light on the origin of these superficial positioned cells, we analysed three further retrograde tracer injections (Figure 2). In one case, the FB injection site was clearly positioned in nucleus reuniens (207#1) (although some track leakage into the AM nucleus could not be completely excluded). In a second case the FB injection was centred in, and restricted to, both nucleus reuniens and the rhomboid nucleus (215#4). Finally, in a third case, an iontophoretic CTB injection targeted the ventral portion of nucleus reuniens but also included a portion of the PaXi and posterior hypothalamic nuclei (215#8). Consistent with the previously described experiments, in the case with the most restricted injection in nucleus reuniens (207#1) the retrograde cell label was limited to the deepest subicular layer (in both dorsal and ventral portions) as well as layer VI in the frontal cortices. In the case with rhomboid involvement of the injection site (215#4) this same labelling distribution was observed, except now very scattered labelling was also present in cortical layer V and superficial subicular cell lamina. Strikingly, compared to this, in the case (215#8) where the tracer deposit in nucleus reuniens extended ventral into the PaXi and posterior hypothalamic nuclei the superficial subicular labelling was considerably denser and the cortical layer V label was, again, much more numerous.

## 4 DISCUSSION

**Figure 13:**
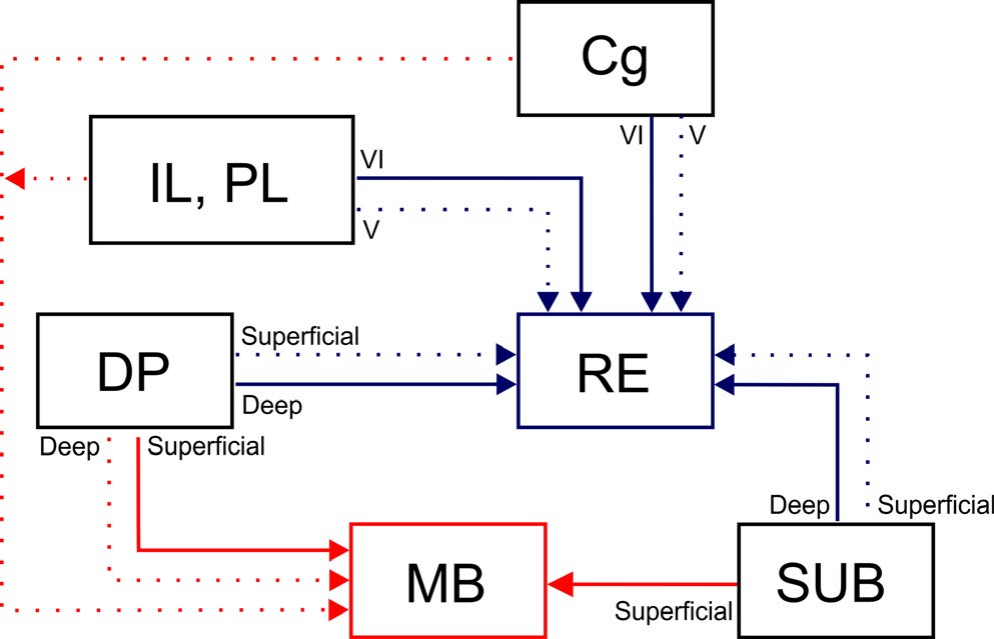
Schematic showing subicular and frontal cortical pathways to nucleus reuniens (blue) and the mammillary bodies (red). Frontal cortical areas densely target nucleus reuniens and these projections originate predominantly from layer VI with minor, additional inputs from layer V. In contrast, a dense projection to the mammillary bodies originates from the dorsal peduncular cortex, predominantly from cells positioned superficial to those cells that project to nucleus reuniens. Further weak inputs to the mammillary bodies originate from the anterior cingulate, infralimbic and prelimbic cortices. The subiculum targets nucleus reuniens predominantly from its deepest lamina, while more superficially positioned cells target the mammillary bodies. Solid lines indicate a dense projections, dashed lines a weak projection. Cg, anterior cingulate cortex; DP, dorsal peduncular cortex; IL, infralimbic cortex; MB, mammillary bodies; PL, prelimbic cortex; RE, nucleus reuniens; SUB, subiculum. [Colour figure can be viewed at wileyonlinelibrary.com]

The principal goal of the present study was to determine the extent of overlap between those hippocampal and frontal neurons that project to the mammillary bodies and nucleus reuniens. In the subiculum there was typically a distinction based on laminar level, such that while the inputs to nucleus reuniens arose from similar anterior‐posterior levels as those to the mammillary bodies, they predominantly originated in deeper cells. Furthermore, while many of the subiculum projections to nucleus reuniens arose from polymorphic cells, the dense projections to the mammillary bodies consistently arose from pyramidal cells. Although the distribution of frontal cells that project to these two diencephalic sites was more complex, it was again found that the populations remained distinct, often separated by cortical layers. When other cortical areas were examined the conclusion remained that these two diencephalic nuclei receive inputs from distinct neurons, even when the cell populations overlap (Figures 10, 11 and 13).

Within the hippocampus, the present study focussed on the subiculum, as it projects to both nucleus reuniens and the mammillary bodies. Despite its rather homogeneous cytoarchitectonic appearance, the connectivity of the rodent subiculum is highly complex (Aggleton & Christiansen, 2015; Ishizuka, 2001; Naber & Witter, 1998; Witter et al., 1990). Many subiculum output pathways can be distinguished according to four distinct zones, based on their temporal – septal (anterior – posterior) and proximal – distal (transverse) regions of origin (Naber & Witter, 1998; Witter, 2006; Witter et al., 1990). For some connections, there is also a laminar separation between distinct subicular outputs (Ishizuka, 2001; Wright, Erichsen, Vann, O'Mara, & Aggleton, 2010). While these topographies help to separate many efferent pathways, some subiculum efferents have collateral projections to more than one site (Calderazzo; Donovan & Wyss, 1983; Kinnavane; Swanson, Sawchenko, & Cowan, 1981). Two relevant examples concern the subicular projections to the mammillary bodies, some of which collateralize to reach the entorhinal cortices (Donovan & Wyss, 1983; Roy et al., 2017) or the retrosplenial cortex (Kinnavane et al., 2018).

Previous research shows that the hippocampal projections to nucleus reuniens and the mammillary bodies arise from across the proximal ‐ distal extent of both the dorsal and ventral subiculum (Allen & Hopkins, 1989; McKenna & Vertes, 2004; Naber & Witter, 1998; Witter et al., 1990). This same overall pattern was seen in the present study, although at septal levels, the projections to nucleus reuniens tended to be densest from proximal portions (see Figures 4e–g, 6 and 7a). Even so, the inputs to nucleus reuniens were consistently matched by projections to the mammillary bodies from the corresponding proximal – distal location (Christiansen et al., 2016; Shibata, 1989). One difference, however, is that a weak, additional projection arises from CA1 to innervate nucleus reuniens (Cenquizca & Swanson, 2006; McKenna & Vertes, 2004). Arguably, a more significant difference, concerns their respective levels of origin, as the large majority of inputs to nucleus reuniens arose from deeper cells in the subiculum. This same pattern of segregation is also seen for the subiculum inputs to the anterior thalamic nuclei and the mammillary bodies (Christiansen et al., 2016; Wright et al., 2010).

Herkenham (1978) reported that nucleus reuniens receives afferents from both deep and superficial subicular lamina that adhere to a medio‐lateral topography, such that the deep subiculum principally projects to the lateral reuniens whereas the medial reuniens receives inputs from all subicular layers. While we also observed retrogradely labelled cells in the superficial layers of the subiculum, a comparison between injection sites suggested that these more superficial cells were associated with those injections that extended beyond nucleus reuniens, i.e. that some superficial cells project to nearby nuclei and not to nucleus reuniens (but see Varela et al., 2014). It is however, important to be cautious as our restricted injections within reuniens could not cover the entire nucleus. At the same time, anterograde viral tracing from the subiculum showed appreciable inputs to the submedius nucleus of the thalamus, alongside much weaker label in the rhomboid nucleus, i.e. sites adjacent to nucleus reuniens. Other structures adjacent to nucleus reuniens were also labelled, including the paraxiphoid nucleus and posterior hypothalamus. Clearly, it would be of value to define the laminar sources of these additional projections from the hippocampus. Lastly, a dorso‐ventral topography has been described such that the dorsal subiculum targets dorsal reuniens and the ventral subiculum targets the ventral reuniens (Herkenham, 1978; but see McKenna & Vertes, 2004). This description agrees with our observation of mainly dorsal subicular label in some cases with dorsal reuniens tracer injections, as well as finding that the anterograde transport of viral markers from the dorsal subiculum often showed a preference for dorsal reuniens.

The present study reinforces the notion that the subiculum is topographically organized along all three axes. The resulting heterogeneity of subiculum cells might, for example, relate to the presence of multiple types of subiculum cells with different spatial properties (Brotons‐Mas; Cembrowski et al., 2018). The present findings also suggest that the cellular layer of the rat subiculum, which is sometimes regarded as a single lamina (Kloosterman, Witter, & Van Haeften, 2003) might be better subdivided to reflect this difference in projection sites. Such a division would match the greater presence of non‐pyramidal cells in the deepest layer of the rat subiculum (Ishihara & Fukuda, 2016), some of which project to nucleus reuniens. Interestingly, clearer lamina differences within the subiculum are seen in primate brains (Ding, 2013; Lorente de Nó, 1934), where there is a distinct deep layer of polymorphic cells, which again provides thalamic inputs (Christiansen et al., 2016).

The second goal of the present study was to re‐examine the distribution of frontal inputs to the mammillary bodies in the light of apparent inconsistencies in previous descriptions of their precise source (Allen & Hopkins, 1989; Hurley et al., 1991; Shibata, 1989; Vertes, 2004). A particular concern is that those studies involving retrograde tracers might unintentionally include frontal projections to the adjacent supramammillary nucleus (Allen & Hopkins, 1989; Shibata, 1989). This potential confound may then explain the apparent increased distribution of frontal projections to the mammillary bodies that is reported in retrograde tracer studies, when compared with the outcome of anterograde tracer injections in frontal cortices (Allen & Hopkins, 1989; Hayakawa et al., 1993; Hurley et al., 1991; Sesack et al., 1989; Shibata, 1989; Vertes, 2004).

Previous studies have reported a topographical organization such that frontal inputs to the mammillary bodies originate from the dorsal peduncular cortex while the infralimbic cortex specifically targets the supramammillary nucleus (Allen & Hopkins, 1989; Hayakawa et al., 1993; Hurley et al., 1991; Shibata, 1989; Takagishi & Chiba, 1991; Vertes, 2004). Our combined anterograde and retrograde datasets corroborate descriptions of relatively dense projections from the dorsal peduncular cortex to the mammillary bodies (Hayakawa et al., 1993; Hurley et al., 1991). However, our data also indicate that some inputs from both the infralimbic and prelimbic cortices can reach the mammillary bodies. Particularly, in two cases with injection sites in the ventral portion of the mammillary bodies, slightly denser retrograde labelling was seen in these cortical areas (including layer VI).

It has previously been reported that the anterior cingulate cortex specifically targets the supramammillary nucleus and not the mammillary bodies (Hayakawa et al., 1993). However, we found evidence of a sparse projection to the medial mammillary nucleus. Some uncertainty also surrounds the question of whether a projection from the subiculum targets the supramammillary nucleus (Canteras & Swanson, 1992; Kishi et al., 2000; Witter et al., 1990). Our anterograde tracing data, in agreement with Hayakawa et al. (1993), indicate that the dorsal subiculum has an extremely weak, additional input to the supramammillary nucleus, which isovershadowed by the far denser inputs to the mammillary bodies. This distinction is significant as it largely removes the potential confound of retrogradely labelled subiculum cells reflecting supramammillary, rather than mammillary body, tracer uptake.

Regarding frontal cortical afferents to nucleus reuniens, these have previously been described as involving layer V, besides the denser layer VI projections (McKenna & Vertes, 2004). It is, therefore, likely that the cortical layer V cells we observed in cases with less restricted reuniens injections (Figure 11) involve a portion of the projections to nucleus reuniens. Importantly, however, this cell population, although partly intermixed, remained distinct from the projections to the mammillary bodies. Again, in the dorsal peduncular cortex, an overall laminar separation was present for the cell populations reaching the mammillary bodies and nucleus reuniens. As was the case with the intermixed cell populations in layer V, in the few cases where cells reaching both nucleus reuniens and the mammillary bodies were intermixed in either layer VI or the deep dorsal peduncular cortex, these cell populations remained distinct.

Within the frontal region, the dorsal peduncular cortex was the most interlinked with the mammillary bodies. The peduncular projections appear focussed in the dorsal mammillary bodies, including the median mammillary nucleus. This same mammillary nucleus has dense projections that target the interanteromedial thalamic nucleus (Shibata, 1992). The same thalamic nucleus then provides much of the anterior thalamic nuclei inputs to the prelimbic and infralimbic cortices (Hoover & Vertes, 2007). This connectivity has potential relevance for the recent suggestion that projections from the subiculum to the mammillary bodies play a role in autonomic responses following fear‐place conditioning (Roy et al., 2017; see also Krieckhaus, 1964). Both the dorsal peduncular and the infralimbic cortex are highly connected to autonomic areas and have been considered to be viscero‐motor areas involved in the control of autonomic function (Frysztak & Neafsey, 1991, 1994; Heidbreder & Groenewegen, 2003).

The present findings have other functional implications. The first concerns the more consistent importance of the mammillary bodies, than nucleus reuniens, for spatial memory tasks that are hippocampal sensitive (Sziklas & Petrides, 1998; Vann & Aggleton, 2003), despite the many projections from nucleus reuniens to the hippocampus (Dolleman‐Van Der Weel & Witter, 1996; Varela et al., 2014; Vertes; Wouterlood, Saldana, & Witter, 1990). This functional difference partly reflects the very extensive subiculum inputs to the mammillary bodies (but see Vann, Erichsen, O'Mara, & Aggleton, 2011), which considerably outnumber the hippocampal inputs to nucleus reuniens. For related reasons, the nature and any redundancy within the information conveyed from the subiculum to the mammillary bodes is of particular interest (Dillingham, Frizzati, Nelson, & Vann, 2015; Kinnavane et al., 2018; Roy et al., 2017). Meanwhile, the influence of nucleus reuniens on learning and memory often appears to combine both frontal and hippocampal processes, e.g. the switching of strategies to solve spatial tasks (Dolleman‐van der Weel et al., 2009; Viena, Linley, & Vertes, 2018). At the same time, the much greater frontal connections of nucleus reuniens, e.g. with prelimbic cortex, point to a broader role in learning and memory that is far less tied to spatial information than the mammillary bodies (Ito et al., 2015; Xu & Sudhof, 2013).

Reflecting these subtle differences in function are the separate subiculum cell populations that reach different diencephalic sites (see also Wright, Vann, Erichsen, O'Mara, & Aggleton, 2013; Wright et al., 2010). Consistent with this pattern, only a very limited number of subiculum cells project to both nucleus reuniens and medial frontal areas (Varela et al., 2014). The clear impression from this complex array of adjacent, but separate, projections from the hippocampus to the medial diencephalon is that they may provide high‐resolution signals capable of discriminating different aspects of spatial information (Jankowski et al., 2014, 2015). Thus, for subiculum cells innervating the thalamus, the overarching principle appears to be that the neurons have only one target site (see also Donovan & Wyss, 1983; Namura, Takada, Kikuchi, & Mizuno*,* 1994; Wright et al., 2013). In contrast, there is an appreciable population of reuniens efferents that collateralize to reach both medial frontal and hippocampal areas (Varela et al., 2014), indicative of a more diffuse executive role.

## CONFLICT OF INTEREST

The authors declare no conflict of interest.

## AUTHOR CONTRIBUTIONS

M.L.M., tracer injections, data analysis, and ms. preparation; E.A., histological analyses; A.J.D.N. and C.M.D. additional tracer injections; S.M.O'M. funding, ms. preparation; J.P.A. funding, experimental design, ms. preparation.

## Data Availability

Available on request to the communicating author.
