# Supplementary material for: Separate cortical and hippocampal cell populations target the rat nucleus reuniens and mammillary bodies
Source: Eur J Neurosci. 2019 Feb 21;49(12):1649–72. doi: 10.1111/ejn.14341 (PMC6618334; doi:10.1111/ejn.14341)
Supplement: Supplementary file 1 [file EJN-49-1649-s001.docx]

| Separate cortical and hippocampal cell populations target the rat nucleus reuniens and mammillary bodies | |
| --- | --- |
|  | Mathiasen, Mathias; Amin, Eman; Nelson, Andrew; Dillingham, Christopher; O'Mara, Shane; Aggleton, John |

_____________________________________________________________________________________

| Review timeline: | Submission date: 25/01/18  Editorial Decision: Major revision  Revision Received: 07/06/18  Editorial Decision: Minor revision  Revision Received: 08/10/18  Editorial Decision: Minor Revision  Revision Received: 28/11/18  Accepted: 04/12/18 |  |
| --- | --- | --- |
|  |  |  |

Editor: Helen Barbas

Reviewer 1: Miles Herkenham

Reviewer 2: Robert Vertes

Reviewer 3: Anonymous

_____________________________________________________________________________________

1st Editorial Decision	21/02/18

_____________________________________________________________________________________

Separate cortical and hippocampal cell populations target the rat nucleus reuniens and mammillary bodies 

Dear Dr. Mathiasen, 

Your manuscript has been reviewed by external reviewers as well as by the Section Editor,  Dr. Helen Barbas, and ourselves.  The reviews collectively indicate that your experiments generated new and important information.  However, there are several issues that need to be clarified/resolved before we can consider your manuscript further for publication in EJN. 

As you can see, the Reviewers provided supportive statements about your study and the overall conclusions, but they also raised important issues and the need for extensive revision. The Reviewers offered a series of thoughtful comments to help increase the precision of the findings and justify their interpretation. One of the common themes in the reviews is the need to better place the findings in the context of the literature on this topic. Confirmatory data can be useful in the field, but it is important to acknowledge their extent, and indicate whether, and how, your findings extend knowledge in a useful direction. It is important to clarify the overall number of cases used, and whether a sufficient number was included for the analyses. It appears that there may not be enough cases with confined injection sites to reach definitive conclusions, but this will not be clear until you reconstruct and illustrate the injection sites fully. This analysis will help decide whether additional cases are needed.  Careful reconstruction of the injection sites will also clarify whether there are differences in the connections of the medial versus the lateral aspect of reuniens, in view of the classic literature on this topic. Some of the data, including anterograde labeling and projection from the mammillary bodies, are too sketchy and need to be expanded substantially. There is need for rigorous quantitative analyses to determine whether the goals set for the study were achieved, including the extent of projections from prefrontal areas, and to help justify the major conclusions for all aspects of the study. There is consensus for the need of further information on methodology. The quality of the figures needs to be improved, including the brightness of the fluorescence photomicrographs, which could be enhanced using confocal microscopy and deconvolution. It is also necessary to increase the precision of the terminology used, including clarification of the border of the dorsal subiculum from the postsubiculum, and to avoid using broad terms, such as ‘frontal’. The Reviewers raised many other useful points to help clarify the methods, disambiguate long sentences, and overall increase the precision of Results and Discussion. 

When revising the manuscript, please embolden or underline major changes to the text so they are easily identifiable and DO NOT leave 'track change' formatting marks in your paper.  Please ensure that you provide a text and a figure file for the Graphical Abstract (as detailed in the instructions below).  When carrying out your revisions please refer to the checklist below and visit the EJN author guidelines at www.ejneuroscience.org 

When finalized, please upload your complete revised manuscript onto the website, as a Word file (.doc, or .docx).  Please also ensure that a complete set of tables and figures is included as separate files, even if these have not changed from the originals.  At this stage it is necessary to provide high resolution figures.  Please see important instructions below. 

Please go into https://mc.manuscriptcentral.com/ejn - Author Centre - manuscripts with decisions where you will find a 'create a revision' link under 'actions'. We ask that you please indicate the way in which you have responded to the points raised by the Editors and Reviewers in a letter. Please upload this response letter as a separate Word (.doc or PDF) file using the file designation "Authors' Response to Reviewers" when uploading your manuscript files.  Please DO NOT submit your revised manuscript as a new one.  Also, please note that only the  Author who submitted the original version of the manuscript should submit a revised version. 

If you are able to respond fully to the points raised, we would be pleased to receive a revision of your paper within 12 weeks. 

Thank you for submitting your work to EJN. 

Kind regards, 

Paul Bolam & John Foxe 
co-Editors in Chief, EJN 


Reviews: 

Reviewer: 1 

Comments to the Author 
In this study, retrograde fluorescent tracers were placed in mammillary bodies and nucleus reuniens, and cells of origin were mapped in several cortical areas.  The major findings were that there are very few double-labeled cells and that the thalamic projection arose from deeper lying cortical cells than the hypothalamic projection.  Some anterograde tracers (not shown) supported the conclusions and pinpointed the localizations.  The findings are for the most part supportive of the rather large literature on the topic, and they are nicely discussed in thorough and scholarly Results and Discussion sections. 
Previous studies, as cited, argue that subicular cells do not collateralize their projects to distal targets, so the finding of lack of double label is in accordance with prior conclusions.  The data are also consistent with the general plan of deep cortical (including subicular) cells projecting to the thalamus and more superficial cells projecting elsewhere. 
1. One point of concern is that Herkenham, 1978; Witter et al., 1990; McKenna & Vertes, 2004; and Varela et al., 2014 all show and discuss the observation that cells located at all depths of subiculum send projections to reuniens.  In fact, a topography was suggested by Herkenham (1978) saying that medial reuniens received inputs from all depths whereas lateral reuniens received inputs from only the deepest cells.  There are instances here of reported superficial labeling, shown in Fig. 4 and 5, but the significance is not discussed (and the figures are so dim, the cells cannot really be seen).  This raises the issue of how thorough the entire reuniens was probed in this study.  There is no real discussion or attempt to manage topographies of tracer injections within the two targeted nuclei. 
2. In that regard, the drawings of the tracer injections in Figure 1 make it look like multiple injections were made in each target structure.  It is hard to understand a single injection having such different deposit locations going from rostral to caudal.  In reuniens, for example, one drawing shows the injection placed laterally in the nucleus, and the next drawing shows it on the midline.  The injections also extend for almost a millimeter in the rostral-caudal direction, suggesting more spread in that dimension than the medial-lateral dimension.  This is confusing.  Why weren't the injections made iontophoretically? 
3. There is awkward wording in places.  For example, the Abstract states "Retrograde tracer injections in rats showed how these two diencephalic sites receive projections from separate cell populations, often from adjacent laminar." Doesn't that sentence need to be completed, like with "…from adjacent layers in the same cortical structure"?  Later, "more morphological diverse" should be "more morphologically diverse."  There are other places where the order of wording is inconsistent in a lengthy sentence making comparisons, so that the comparisons are hard to follow. 
4. Methods: the volume injected and rate of injection should be specified for both retrograde and anterograde tracers. 
5. The photomicrographs are almost impossible to see and are blurry.  The images should be collected on a confocal microscope and re-displayed.  If these images are used, major contrast enhancement and brightness boost are needed. 
6. The split images in Fig. 4 add no value.  Only the top three should be displayed, ideally at a larger size (and enhanced). 
7. On page 10, reference to Fig. 9 in description of labeling pattern probably should be to Fig. 8. 


Reviewer: 2 

Comments to the Author 
Using a combination of retrograde (mainly) and anterograde tracers, the authors examined the distribution of projections from the subiculum of the hippocampus and regions of the medial prefrontal cortex to nucleus reuniens (RE) of the midline thalamus and to the mammillary body (MB) – two regions (among other things) involved in spatial working memory (SWM).  They described essentially no overlap in the projections from the subiculum or prefrontal cortices to these two diencephalic sites, indicating separate forebrain circuitries modulating the activity of RE and MB.  The findings significantly point to a segregation of these two diencephalic systems, suggesting that they independently contribute to different (but possibly complementary) aspects of SWM – and other functions.   

The results are important, quite well described (but see below) and convincing.   

Comments: 

1.  The last paragraph of the Introduction discusses injections confined to nuclei vs. those spreading to adjacent sites. This involves methods and should be in the Methods section. 

2.  In the beginning of the Methods section, they indicate that 25 rats were used but further in the Methods and Results they describe the use 25 rats for the retrograde work and another 7 rats for the anterograde tracer study.  What were the total number of rats used?  Further on this, for the retrograde work they mention that 7 rats were eventually used for the analysis (see Fig 1); however, only 2 rats had injections confined to RE and MB, the other 5 with injections spreading outside RE/MB. Are two rats (or two good injections) an adequate sample?   

3.   They need to be more precise with reference to “frontal” areas (e.g, p. 9, “Frontal and other cortical afferents”.  For the most part they examined input to the two sites from the medial prefrontal cortex (dorsal peduncular, infralimbic, prelimbic, anterior cingulate and to some extent the medial orbital cortex). This should be clearly defined; “frontal” is used loosely. 

4.  With apparently one exception, FB was deposited in the RE and CTB in MB. With this in mind, they repeatedly qualify the FB (or CTB) injections by pointing to the location of the injections, e.g., p. 10, “Dense FB labeling (reuniens injections)”. This is not necessary or would suggest to “Dense labeling with reuniens injections ..” 

5.   The results are qualitative and not quantitative. While this may not affect the major findings of two segregated populations of subicular or mPFC cells projecting to RE or MB, perhaps they could be more definitive in describing numbers of double labeled cells – give percentages rather than just stating they were rare or extremely limited.   

6.    At the level of magnification shown, Fig. 1 is too small. Without enlarging the figure (which the reader should not be expected to do) the abbreviations for structures are difficult read; would be much better at the size of the text of the other schematic figures (e.g., Figs 2,8). 

7.   The photomicrographs should be improved, particularly Fig. 3F,J (only a blue background, can’t see individually labeled cells); Fig. 5A,B (can’t see the CTB+ cells resulting from the RE injection) and Fig. 7A-C [can’t see the CTB+ cells in A-C (especially in C) or the FB cells in C]. 

8.    A major issue relates to the anterograde work.  7 anterograde injections were made in the subiculum (3) and mPFC (one in DP and 3 in the anterior cingulate cortex). However, unlike their relatively complete description of the retrograde finding (with the use of schematics and micrographs), no actual data is shown for the anterograde work, including injection sites and patterns of anterograde labeling. Accordingly, without this, they should remove the description of the anterograde findings. The conclusions based on the anterograde work (see Discussion) need to be supported. In addition, aside from the single DP injection, is unclear why the anterograde injections were restricted to the anterior cingulate (AC) cortex, when IL or PL are stronger sources of afferents to RE than AC?   

9.  Similar to pt 8, the section on “mamillary bodies to nucleus reuniens” should be removed (unless supported by actual data).  At present, the findings appear quite observational – an afterthought to the main theme of the report.  The issue of the source of head direction input to RE is certainly important, but the present results are inconclusive; more needs to be done.   

10.  Fig. 9 should be described in the body of the Results. 

11.  There is no need for Table 1, the information contained in it is well described in the text.     

12.  Overall the Discussion is well done, possibly with the exception of the section on DP and subicular influences on MB in autonomic control – quite speculative.       
  


Reviewer: 3 

Comments to the Author 
The manuscript by Mathiasen et al. (EJN-2018-01-25288) described and compared the origins of hippocampal (subicular) and frontal (areas PL, IL and DP) inputs to nucleus reunions (RE) and medial mammillary nucleus (MN) using two traditional retrograde tracers in the same animals. The main finding is that separate cell populations (often separate layers) in the subiculum and in the frontal cortex project to RE and MN. In the subiculum, the cells projecting to RE are mostly located in the deep layer (polymorphic layer) while those projecting to MN mostly in the superficial layer (pyramidal layer). In the frontal cortex, the neurons projecting to MN are mainly located in the superficial layers of the dorsal peduncular cortex (DP), IL and PL while those to RE mainly in the deeper layers (layers V and VI) of the PL and IL. However, almost all these findings were previously reported (see below, points 1 and 2) and thus this manuscript, using two tracers in single animals, mainly confirms the previous findings derived from single tracer studies. 
Point 1: After placing the retrograde tracers in RE, Herkenham (1978, case R2) and McKenna and Vertes (2004) found that the labeled cells were mostly seen in the deep layers (mainly layer 6) of the PL, IL/DP and anterior cingulate area of the frontal cortex, as well as in the deep layer (polymorphic layer) of the subicular cortex. Anterograde tracer studies confirmed these findings (e.g. Hurley et al., 1991; Vertes, 2002; 2004). 
Point 2: After placing the retrograde tracers in the MN, the many labeled cells were mostly seen in the superficial layers (i.e. pyramidal layer) of the subiculum [Shibata (1989), Witter et al. (1990), Ishizuka, 2001; Wright et al. (2010)] while fewer labeled cells (mainly layer 5) were observed in PL, IL/DP (Allen and Hopkins, 1989; Shibata, 1989, Fig .1F). These findings were further confirmed using anterograde tracing experiments (for IL/DP injections: Shibata, 1989; Hurley et al., 1991; Takagishi and Chiba, 1991; Vertes, 2004). 

Specifically, the following major issues need to be addressed: 
1. In the Introduction section, 
(1) In paragraph 3, the authors indicated that they aimed to examine the extent of the frontal cortical projections to the MN. In their results, however, they did not carry out related cell counts and thus are not able to evaluate the extent convincingly. 
(2) In paragraph 4, the authors claimed that an input from the MN (to RE) has not yet been reported (McKenna and Vertes, 2004). In fact, McKenna and Vertes (2004) reported this input (see table 1 and figures). Therefore, the base of the second aims of this manuscript does not exist. 
(3) In paragraph 5, the authors pointed out the challenges of accurate placement of the tracers in target structures without leaking into adjacent regions. However, they only got two cases in which the tracers were accurately targeted (cases 208#9 and 209#3). 

2. In the Results section, 
(1): in the subsection subicular afferents to RE and MN (page 8, for case 208#9), the authors reported that “at septal levels, both tracers labeled cells predominantly within the proximal subiculum, ……”. However, in cases 209#10, in which the tracer FB was limited in MN, the labeled cells were mostly observed within the distal subiculum (i.e. near postsubiculum; see their Fig 5C). How to explain and prove the obvious difference between these two cases (both have MN injections)? How about the case 209#3 and other cases? 

(2) For the cases in which the tracers involved in the adjacent regions (pages 8, 9 and 11), detailed description is not needed because the results are hard to be interpreted. Instead, only the parts related to the goals of the manuscript need to be described, shown and explained. 

(3). Anterograde tracing results are very important for confirming the results from retrograde tracing, especially when many retrograde tracer injections were not limited in the target regions (as in this manuscript). Therefore, it is necessary to show the injection sites and terminal labeling in target structures of the anterograde tracing experiments. 

(4). In Figs. 2-5, the placement of the border between the dorsal subiculum and postsubiculum is obviously different from that in Christiansen et al. (2016).  This suggests a definition issue of the subiculum. 

3. In the discussion section, 
(1). Inconsistence results regarding the proximal and distal distribution of the labeled cells in the subiculum after MN retrograde tracer injections need to be explained with proper evidence. (Refer to 2(1) above) 
(2). The authors claimed that their second goal is to re-examine the distribution of frontal inputs to the MN and that they found some inputs from both the infra- and prelimbic cortices reaching the MN (see last paragraph of page 14).  In fact, the projections from PL, IL/DP to MN were previously reported (see the Point 2 above). 
(3). Regarding the third goal, the authors claimed that no input from the MN (to RE) appears to have been described (page 15, 2nd paragraph). Actually, this input was clearly reported and shown (McKenna and Vertes, 2004, see table 1 and Figs 2I, 6I, 8I). Therefore, the discussion in this paragraph does not make much sense. 
(4) Regarding functional implications of the main findings (last paragraph of page 15), the authors appear to focus on hippocampal-MN or frontal-RE projections, which are the findings of many previous studies (see the Points 1 and 2 above). A new finding of this manuscript maybe the separate cell populations projecting to RE and MN. It is functional implications related to this finding that need to be discussed. 
(5). In the last paragraph, the authors claimed that only the anterior thalamic nuclei (but not the RE) are innervated by mammillary bodies. However, the fact that the Re is also innervated by mammillary bodies (both lateral and medial mammillary nuclei) was shown previously (McKenna and Vertes, 2004, see their table 1 and figures). Additionally, evidence for the existence of ventral/temporal subicular projections to the anterior thalamic nuclei also exists in both rodent and monkey. 

------------------------ ____________________________________________________________________________________

Authors’ Response

_____________________________________________________________________________________

Dear EJN

May I thank you for the detailed reviews and the opportunity to revise our manuscript. Can I first apologise for the lengthy delay in providing this revision, but it became necessary to include new cases to respond to the helpful suggestions for improvement. These new cases involve both anterograde and retrograde tracer injections. We feel that these cases significantly strengthen the various conclusions within the study.

A consequence of providing this further information means that we now have 12, rather than 9, figures for the study. For this reason, Figure 1, which depicts retrograde tracer injection sites, has been extended so that it is now Figures 1 & 2. Meanwhile the new Figure 3 depicts the injections sites of the anterograde tracer cases (as requested). In addition, Figure 11 shows the pattern of termination within the mammillary bodies from an injection in dorsal peduncular cortex.

Finally, we have removed what was the final paragraph in the Discussion. We did this partly because the Discussion had become overlong (as we were asked to add more material) and partly because the topic was slightly tangential to the aims of the present study.

When uploading our figures in high resolution tiff. format the images were far too large for the 100MB total file size constraint at https://mc.manuscriptcentral.com/ejn. Therefore, we have submitted our figures in the JPEG format, which have reduced the image quality. A main complaint for all reviewers were the image resolution. We have solved this issue, but as the figures now are of slightly less quality (compared to tiff files) there is a risk that they will appear of low quality too. Therefore, if the quality of the images are deemed to be insufficient we will be happy to submit our original tiff files.

Below I have reproduced your summary comments as well as the individual comments of the Reviewers. We have provided detailed responses to the latter.

*One of the common themes in the reviews is the need to better place the findings in the context of the literature on this topic. Confirmatory data can be useful in the field, but it is important to acknowledge their extent, and indicate whether, and how, your findings extend knowledge in a useful direction. It is important to clarify the overall number of cases used, and whether a sufficient number was included for the analyses. It appears that there may not be enough cases with confined injection sites to reach definitive conclusions, but this will not be clear until you reconstruct and illustrate the injection sites fully. This analysis will help decide whether additional cases are needed. Careful reconstruction of the injection sites will also clarify whether there are differences in the connections of the medial versus the lateral aspect of reuniens, in view of the classic literature on this topic. Some of the data, including anterograde labeling and projection from the mammillary bodies, are too sketchy and need to be expanded substantially. There is need for rigorous quantitative analyses to determine whether the goals set for the study were achieved, including the extent of projections from prefrontal areas, and to help justify the major conclusions for all aspects of the study. There is consensus for the need of further information on methodology. The quality of the figures needs to be improved, including the brightness of the fluorescence photomicrographs, which could be enhanced using confocal microscopy and deconvolution. It is also necessary to increase the precision of the terminology used, including clarification of the border of the dorsal subiculum from the postsubiculum, and to avoid using broad terms, such as ‘frontal’. The Reviewers raised many other useful points to help clarify the methods, disambiguate long sentences, and overall increase the precision of Results and Discussion.*

Reviews:

**Reviewer: 1**

*1.	One point of concern is that Herkenham, 1978; Witter et al., 1990; McKenna & Vertes, 2004; and Varela et al., 2014 all show and discuss the observation that cells located at all depths of subiculum send projections to reuniens. In fact, a topography was suggested by Herkenham (1978) saying that medial reuniens received inputs from all depths whereas lateral reuniens received inputs from only the deepest cells. There are instances here of reported superficial labeling, shown in Fig. 4 and 5, but the significance is not discussed (and the figures are so dim, the cells cannot really be seen). This raises the issue of how thorough the entire reuniens was probed in this study. There is no real discussion or attempt to manage topographies of tracer injections within the two targeted nuclei.*

The issue of subiculum topography is of considerable interest, especially given how the subiculum projects densely to both targets under investigation. Closely linked is the issue of depth of inputs. We have explained much more explicitly how previous investigations have described such inputs (e.g. Herkenham, 1978; McKenna & Vertes, 2004; and Varela et al., 2014). A related issue is the suggestion (Herkenham, 1978) that dorsal reuniens is targeted by dorsal subiculum whereas the ventral portion of the nucleus is targeted by the ventral subiculum. (However, another topography is suggested in McKenna & Vertes 2004).

While we cannot pretend to give a comprehensive analysis (as our primary focus was on collateral projections), there is a consistent pattern to our findings, whereby more superficial cells are seen when the tracer injections extend beyond nucleus reuniens. (We have, for example added a new section in the Results and drawn data from more cases with reuniens injections.) We have accordingly added more space in the Discussion to this issue. While we could make inferences regarding a dorsal/ventral topography, we do not feel that we have sufficient cases to make a strong conclusion. Lest these should be seen as shortcomings it must be remembered that the

main purpose of the paper is to describe the separation of input to the mammillary bodies and nucleus reuniens, and their detailed, intrinsic topographies.

*2.	In that regard, the drawings of the tracer injections in Figure 1 make it look like multiple injections were made in each target structure. It is hard to understand a single injection having such different deposit locations going from rostral to caudal. In reuniens, for example, one drawing shows the injection placed laterally in the nucleus, and the next drawing shows it on the midline. The injections also extend for almost a millimeter in the rostral-caudal direction, suggesting more spread in that dimension than the medial-lateral dimension. This is confusing. Why weren't the injections made iontophoretically?*

Single injections were made in each site, but using an angled approach to avoid the central sinus. This might explain some confusion over the bolus shape. Tracer spread is rarely symmetrical and can be hard to control. With respect to the spread of tracer we have looked at our injection sites again and we acknowledge that we in some cases might have exaggerated the spread of the tracer along the a-p axis. We have adjusted the plots accordingly. Finally, we have added data from a number of new cases, with injections either in the cortex or nucleus reuniens. In half of these cases the tracer injections were made iontophoretically (although the pattern of results matches that already seen with other delivery methods).

3.	*There is awkward wording in places. For example, the Abstract states "Retrograde tracer injections in rats showed how these two diencephalic sites receive projections from separate cell populations, often from adjacent laminar." Doesn't that sentence need to be completed, like with "…from adjacent layers in the same cortical structure"? Later, "more morphological diverse" should be "more morphologically diverse." There are other places where the order of wording is inconsistent in a lengthy sentence making comparisons, so that the comparisons are hard to follow.*

We thank the Reviewer. We have amended the text as suggested and tried to improve clarity in other places.

*4.	Methods: the volume injected and rate of injection should be specified for both retrograde and anterograde tracers.*

We have included these data in the method section

5.	*The photomicrographs are almost impossible to see and are blurry. The images should be collected on a confocal microscope and re-displayed. If these images are used, major contrast enhancement and brightness boost are needed.*

There is general agreement that the image quality requires improvement. One problem was in the loss of clarity when moving the submission format. However, we have increased the image quality in most figures by adjusting brightness, contrast and intensity. In addition, we have included some additional higher resolution images that should make the individual cells clearly visible (Figs 5,7,10).

*6.	The split images in Fig. 4 add no value. Only the top three should be displayed, ideally at a larger size (and enhanced).*

This figure is now changed exactly as suggested by the Reviewer.

*7.	On page 10, reference to Fig. 9 in description of labeling pattern probably should be to Fig. 8.*

The reference is changed as suggested.

**Reviewer: 2**

*The results are important, quite well described (but see below) and convincing.*

Comments:

*1. The last paragraph of the Introduction discusses injections confined to nuclei vs. those spreading to adjacent sites. This involves methods and should be in the Methods section.*

The last paragraph has been edited, although we have retained the explanation for using both anterograde and retrograde tracing methods.

*2. In the beginning of the Methods section, they indicate that 25 rats were used but further in the Methods and Results they describe the use 25 rats for the retrograde work and another 7 rats for the anterograde tracer study. What were the total number of rats used? Further on this, for the retrograde work they mention that 7 rats were eventually used for the analysis (see Fig 1); however, only 2 rats had injections confined to RE and MB, the other 5 with injections spreading outside RE/MB. Are two rats (or two good injections) an adequate sample?*

First, we apologise for any confusion over the rat numbers. We have revised the ms to be completely clear. In the original Method section we included a number of animals that we did not describe in the Results section. These animals had fluorogold injection in reuniens but were excluded from the final analyses since the injections leaked into the third ventricle. This led to confusing label patterns. We have now omitted the cases completely from the paper. At the same time, we have added five new cases: two with anterograde tracers (both with two injections) in cortex, three with single retrograde tracer injection in thalamus (including case 207#1 with the injection seemingly confined to nucleus reuniens).

*3. They need to be more precise with reference to “frontal” areas (e.g, p. 9, “Frontal and other cortical afferents”. For the most part they examined input to the two sites from the medial prefrontal cortex (dorsal peduncular, infralimbic, prelimbic, anterior cingulate and to some extent the medial orbital cortex). This should be clearly defined; “frontal” is used loosely.*

In the Introduction we now define how the term ‘frontal cortices’ is used, while being more specific in other locations in the text.

4. With apparently one exception, FB was deposited in the RE and CTB in MB. With this in mind, they repeatedly qualify the FB (or CTB) injections by pointing to the location of the injections, e.g., p. 10, “Dense FB labeling (reuniens injections)”. This is not necessary or would suggest to “Dense labeling with reuniens injections ..”

We have accordingly reduced the references to the nuclei in the sections where we describe injections that are confined the nucleus reuniens and the mammillary bodies.

*5. The results are qualitative and not quantitative. While this may not affect the major findings of two segregated populations of subicular or mPFC cells projecting to RE or MB, perhaps they could be more definitive in describing numbers of double labeled cells – give percentages rather than just stating they were rare or extremely limited.*

We have now added cell counts to give a much clearer perspective on the ratios of single and double-labelled cells. In all cases with combined retrograde tracer injections we manually counted the cells in the subiculum (mean data now provided). In the two cases with confined injection sites we only observed a single double-labelled cell in one of the cases. We did not, however, quantify the frontal label as i) again, it was rare, ii) there remains the possibility that it might be affected supramamillary nucleus uptake (which would have little impact on subiculum counts).

*6. At the level of magnification shown, Fig. 1 is too small. Without enlarging the figure (which the reader should not be expected to do) the abbreviations for structures are difficult read; would be much better at the size of the text of the other schematic figures (e.g., Figs 2,8).*

We have enlarged Figure 1 so that it now fill up two pages. For this reason it has how become Figures 1 and 2.

*7. The photomicrographs should be improved, particularly Fig. 3F,J (only a blue background, can’t see individually labeled cells); Fig. 5A,B (can’t see the CTB+ cells resulting from the RE injection) and Fig. 7A-C [can’t see the CTB+ cells in A-C (especially in C) or the FB cells in C].*

Please see corresponding remarks to Reviewer 1.

*8. A major issue relates to the anterograde work. 7 anterograde injections were made in the subiculum (3) and mPFC (one in DP and 3 in the anterior cingulate cortex). However, unlike their relatively complete description of the retrograde finding (with the use of schematics and micrographs), no actual data is shown for the anterograde work, including injection sites and patterns of anterograde labeling. Accordingly, without this, they should remove the description of the anterograde findings. The conclusions based on the anterograde work (see Discussion) need to be supported. In addition, aside from the single DP injection, is unclear why the anterograde injections were restricted to the anterior cingulate (AC) cortex, when IL or PL are stronger sources of afferents to RE than AC?*

Rather than remove these cases, we have added others cases with anterograde tracers. As part of this process, we have plotted the injection sites for anterograde tracers in frontal cortex and provided a new figure (Fig. 11) that illustrate the fibre innervation of the mammillary bodies by the dorsal peduncular cortex. The four new anterograde tracer injections are placed in the IL and PL cortices as suggested. These new data confirm the initial conclusion that the mammillary bodies are particularly targeted by the dorsal peduncular cortex.

*9. Similar to pt 8, the section on “mamillary bodies to nucleus reuniens” should be removed (unless supported by actual data). At present, the findings appear quite observational – an afterthought to the main theme of the report. The issue of the source of head direction input to RE is certainly important, but the present results are inconclusive; more needs to be done.*

Indeed, for this reason we have removed the relevant section in the Introduction (paragraph 4) and in the Results section. The presence of mammillary body inputs to nucleus reuniens is now only briefly mentioned in the Discussion.

*10. Fig. 9 should be described in the body of the Results.*

We apologise that we omitted reference to Fig. 9 (now Fig. 12). As this is a summary figure it is now referenced in the Discussion.

*11. There is no need for Table 1, the information contained in it is well described in the text.*

We have accordingly removed the Table.

*12. Overall the Discussion is well done, possibly with the exception of the section on DP and subicular influences on MB in autonomic control – quite speculative.*

We agree that this part of the Discussion is speculative, but we feel it provides a link to an older literature on mammillary body lesions and aversive conditionings (e.g. Kriekhaus, 1964 – now cited). We have additionally pointed out the particular link with the median mammillary bodies, which in turn projects to the interoanteromedial nucleus of the thalamus. This same thalamic nucleus provides much of the anterior thalamic nuclei inputs to the prelimbic and infralimbic cortices (Hoover & Vertes, 2007 - now cited).

Reviewer: 3

Specifically, the following major issues need to be addressed:

1.	In the Introduction section,

*(1)	In paragraph 3, the authors indicated that they aimed to examine the extent of the frontal cortical projections to the MN. In their results, however, they did not carry out related cell counts and thus are not able to evaluate the extent convincingly.*

We have now included quantitative data for the subiculum where we compare the two cell populations. For reasons explained above, we have not carried out corresponding cell counts for the frontal cortex afferents from the mammillary bodies. As the cell counts were manual, and not stereological, they provide relative rather than absolute cell numbers.

*(2) In paragraph 4, the authors claimed that an input from the MN (to RE) has not yet been reported (McKenna and Vertes, 2004). In fact, McKenna and Vertes (2004) reported this input (see table 1 and figures). Therefore, the base of the second aims of this manuscript does not exist.*

We can only apologise for this oversight. Herkenham (1978) did not describe this connection but McKenna & Vertes (2004) identified it in some of their figures and in Table 1. We have accordingly deleted para 4 of the Introduction and removed the section dealing this issue in the Results. While the Discussion retains a brief mention of this connection, the extensive paragraph devoted to this issue has now been deleted.

*(3)	In paragraph 5, the authors pointed out the challenges of accurate placement of the tracers in target structures without leaking into adjacent regions. However, they only got two cases in which the tracers were accurately targeted (cases 208#9 and 209#3).*

To add further support to our conclusions we have added data from two further cases with anterograde tracers (Fig. 3) and three with retrograde tracers (Fig. 2). With respect to the issue of double injections in both sites, there were other cases (more now mentioned in the text) that support the pattern seen in the original two cases.

2.	In the Results section,

*(1): in the subsection subicular afferents to RE and MN (page 8, for case 208#9), the authors reported that “at septal levels, both tracers labeled cells predominantly within the proximal subiculum, ……”. However, in cases 209#10, in which the tracer FB was limited in MN, the labeled cells were mostly observed within the distal subiculum (i.e. near postsubiculum; see their Fig 5C). How to explain and prove the obvious difference between these two cases (both have MN injections)? How about the case 209#3 and other cases?*

Based on the limited numbers of cases we do not describe these topographies. Importantly, the topographical organization of the mammillary body afferents from subiculum has been described in previous studies (e.g. Shibata, 1989; Christiansen et al., 2016). These data show that the mammillary bodies receive projections from both proximal and distal portion but that this depends in precise position of the injection site. Therefore, we include this in the Discussion but we do not claim to add any new information to the question.

*(2) For the cases in which the tracers involved in the adjacent regions (pages 8, 9 and 11), detailed description is not needed because the results are hard to be interpreted. Instead, only the parts related to the goals of the manuscript need to be described, shown and explained.*

We have condensed these sections but feel that they have a clear bearing on the issue of whether superficial subiculum cells project to nucleus reuniens. For this same reason, we have added a section at the end of the Results that specifically deals with this issue. (See Reviewer 1 point 1).

*(3). Anterograde tracing results are very important for confirming the results from retrograde tracing, especially when many retrograde tracer injections were not limited in the target regions (as in this manuscript). Therefore, it is necessary to show the injection sites and terminal labeling in target structures of the anterograde tracing experiments.*

This information is now added, as well as additional cases being provided.

*(4). In Figs. 2-5, the placement of the border between the dorsal subiculum and postsubiculum is obviously different from that in Christiansen et al. (2016). This suggests a definition issue of the subiculum.*

We thank the reviewer as careful inspection shows that the border between dorsal subiculum and postsubiculum was indeed slightly in error as it did not take into account the way in which subiculum cells form a curved border. New Figures 4, 6, and 7 have been revised accordingly.

*3.	In the discussion section,*

*(1). Inconsistence results regarding the proximal and distal distribution of the labeled cells in the subiculum after MN retrograde tracer injections need to be explained with proper evidence. (Refer to 2(1) above)*

Please see our response above.

*(2). The authors claimed that their second goal is to re-examine the distribution of frontal inputs to the MN and that they found some inputs from both the infra- and prelimbic cortices reaching the MN (see last paragraph of page 14). In fact, the projections from PL, IL/DP to MN were previously reported (see the Point 2 above).*

Our original text made it clear that such projections had been reported in some but not all studies, hence, the need to re-examine this issue. Of particular concern was whether retrograde tracer studies might have involved the supramammillary nucleus.

*(3). Regarding the third goal, the authors claimed that no input from the MN (to RE) appears to have been described (page 15, 2nd paragraph). Actually, this input was clearly reported and shown (McKenna and Vertes, 2004, see table 1 and Figs 2I, 6I, 8I). Therefore, the discussion in this paragraph does not make much sense.*

This paragraph has been removed.

*(4) Regarding functional implications of the main findings (last paragraph of page 15), the authors appear to focus on hippocampal-MN or frontal-RE projections, which are the findings of many previous studies (see the Points 1 and 2 above). A new finding of this manuscript maybe the separate cell populations projecting to RE and MN. It is functional implications related to this finding that need to be discussed.*

We thank the reviewer for this point and have accordingly added a new paragraph in the Discussion.

(*5). In the last paragraph, the authors claimed that only the anterior thalamic nuclei (but not the RE) are innervated by mammillary bodies. However, the fact that the Re is also innervated by mammillary bodies (both lateral and medial mammillary nuclei) was shown previously (McKenna and Vertes, 2004, see their table 1 and figures). Additionally, evidence for the existence of ventral/temporal subicular projections to the anterior thalamic nuclei also exists in both rodent and monkey.*

As already explained in this response letter, we accept this point and the Discussion amended appropriately.

2^nd^ Editorial Decision	05/09/18

_____________________________________________________________________________________

Separate cortical and hippocampal cell populations target the rat nucleus reuniens and mammillary bodies 

Dear Dr. Mathiasen, 

Your revised manuscript was re-evaluated by external reviewers as well as by the Section Editor, Dr. Helen Barbas and ourselves.  We are pleased to inform you that we expect that it will be acceptable for publication in EJN following further revision and possible re-review. 

The Reviewers appreciated the revisions, which have improved the manuscript. They have also pointed to several remaining points that need to be addressed, as detailed in the reviews. There are no figure legends in the uploaded manuscript, these should be included in the main text. A Table to list the injection sites and key findings would help the readability of Results. References to figures in the text should be in order, and identified by panel in Results. It is necessary to go over the manuscript carefully to correct grammatical errors and disambiguate the meaning. The last paragraph of discussion, in particular, needs work. 

When revising the manuscript, please embolden or underline major changes to the text so they are easily identifiable and please don't leave 'track change' formatting marks in your paper.  At this stage, please provide text and a figure file for the Graphical Abstract.  When carrying out your revisions please refer to the checklist below and visit the EJN author guidelines at www.ejneuroscience.org 

When finalised, please upload your complete revised manuscript onto the website as a Word (.doc or .docx), or .rtf file.  Please also ensure that a complete set of tables and figures is included as separate files, even if these have not changed from the originals.  At this stage it is necessary to provide high resolution figures.  Please see important instructions below. 

Please go into https://mc.manuscriptcentral.com/ejn - Author Centre - manuscripts with decisions where you will find a 'create a revision' link under 'actions'. We ask that you please indicate the way in which you have responded to the points raised by the Editors and Reviewers in a letter. Please upload this response letter as a separate Word (.doc) file using the file designation "Authors' Response to Reviewers" when uploading your manuscript files. Please DO NOT submit your revised manuscript as a new one.  Also, please note that only the  Author who submitted the original version of the manuscript should submit a revised version. 

If you are able to respond fully to the points raised, we shall be pleased to receive a revision of your paper within 30 days. 

Thank you for submitting your work to EJN. 

Best wishes, 

Paul Bolam & John Foxe 
co-Editors in Chief, EJN 

Reviews: 

Reviewer: 1 

Comments to the Author 
The revised version of the paper seems to addresses the reviewers’ concerns, though in some places the additional text is ragged and long-winded, and I lose the thread when reading it.  The final uploaded version has all the figures (almost right) but no figure captions, so I will assume they are appropriate. 

Minor points: 
1. Page 26, discussion: “Although the distribution of frontal cells that project to these two diencephalic sites was more complex, it was again found that the populations remained distinct, often separated by laminar.”  The sentence cannot end with an adjective. 
2. Fig. 11 D-F images are not mentioned in the text.  I’m not sure what the point was of adding Fig. 11 and the text in “anterograde tracer injections in frontal cortices” pp 15-16.  The Discussion about projection to MMMB from DP and IL on p. 19 doesn’t refer to the data and doesn’t really say anything, and I don't understand the point of the added text.  It doesn’t help that I don’t have a figure caption for Fig. 11.  The contrast should be enhanced in the images in D-E.  I can hardly see the light labeling. 
3. The new text on Additional retrograde tracer injections (P. 25) needs editing for correct use of modifiers (note underlining of fixed words): confined injections also labelled layer V relatively densely;  “labelling was considerably denser” 
4. Disc p. 17 “collaterise” should be “collateralize” 
5. The figure quality is improved but could still be better—more contrast enhance mainly would help. 


Reviewer: 2 

Comments to the Author 
The authors made significant improvements in the manuscript but some issues still need to be addressed. 

Comments: 

1.   Although the expanded Figs 1 and 2 are helpful, it is still very difficult to separate each of the retrograde cases (or the patterns of labeling resulting from them) in that most of the findings are descriptive with only limited examples of actual patterns of labeling: schematics or micrographs.  It certainly also does not help that cases are designated, for example, 207#2, 207#4 or 207#9, etc.  What is very much needed for the retrograde cases is a Table listing each of the injections and their main subicular and cortical inputs – to thereby allow for comparisons across cases. This is particularly needed for cortical afferents as sites projecting to reuniens and MB are numerous and widely distributed.   

2.  They indicate that it was possible to differentiate MB from SUM inputs on the basis and selective and differential brainstem afferents to each site: to MB from Gudden’s tegmental nucleus and to SUM from the laterodorsal tegmental nucleus.  It is questionable if this is truly the case or if these criteria were actually used – no data is shown. For instance, as was indicated (p. 14) case 207#7 appears to significantly involve the SUM but virtually no labeled cells were observed in LDT. Unless substantiated, reference to Gudden’s and LDT this should be removed.       

3.  It was important to make anterograde injections (3 injections) in the subiculum to in part determine whether the SUB projects to the SUM – which apparently it does not.  However, no data is shown for this: schematics/micrographs for injection sites or terminal labeling. It would be valuable to include the actual data. Also, what was the pattern of labeling in RE with SUB injections -- and did labeling extend to RH.  Finally, if the subicular injections were layer specific this would significantly support their retrograde findings of deep SUB projections to RE and superficial ones to MB. 

4.   The panels on the figures are nicely labeled (A, B, C, etc) but in the text of Results the figures are most often referred to as a whole (e.g., Fig 7) without specific reference to individual panels.  This is particularly bothersome on pgs. 12 and 13 when referring to Fig. 9 – and not specifying the individual panels. Also it is best to avoid the use “former” and “latter” as was often done in the manuscript. 

5.   They reported (p. 14) that they did not observe any retrogradely-labeled cells in the frontal cortex with case 207#9).  But examination of the FB injection in this case (207#9) shows it to be essentially equivalent to that of case 208#9 -- which exhibited robust retrograde cell labeling throughout the prefrontal cortex (Fig. 9).  What was the difference in the two injections? This needs to be addressed.   

6.  The section of the Results on indusium griseum (IG) (p. 13) is confusing and difficult to follow.  This is partly due to interspersing dorsal and ventral aspects of IG as well as other cortical labeling when describing IG.  For instance, what does “In both cases” (first sentence of the 2nd para on p. 13) refer to. Or “For both cases” – first sentence of the last para on p. 13. See also pts. 1 and 4 above.   

7.  Eight anterograde tracer injections were made in regions of the PFC (Fig. 3). In the first instance, Fig. 3 should appear before Fig. 11. Apparently, the main reason for including the anterograde cases was to show that DP injections gave rise to terminal labeling in medial MB and not in SUM and also that PFC injections that avoided IG produced sparse labeling in MB? Did the dorsal peduncular injections produce labeling in RE; this should at least be discussed. Additionally, the anterograde PFC injections would be expected to yield valuable information on PFC projections to the ventral midline thalamus (or to SUM) – possibly confirming the findings of previous reports. This data may not be important to include here, but the issue should be addressed. 

8.  The summary of the interconnections (bottom of Fig. 11) should probably be a separate figure. 

9.  Would replace the word “frequently” in the Abstract with “most often”.   

In summary, the main findings of the report regarding differential subicular and PFC input to MB and RE are solid, convincing and important.  The above comments are for the most part relatively minor with the possible exception of points 1 and 3. 

------------------------

_

____________________________________________________________________________________

Authors’ Response

_____________________________________________________________________________________

05-Sep-2018

Manuscript Number:EJN-2018-01-25288.R1

Separate cortical and hippocampal cell populations target the rat nucleus reuniens and mammillary bodies

Dear EJN

We have carefully read the suggestions of the referees as well as those of the Section Editor and your own helpful comments. We feel that we have addressed all of these comments. These changes are described below, where each comment is also provided. Edits to the text are underlined. In addition, we have added a Table describing the various injection sites (as recommended) as well as two new figures. One of these figures displays the relative patterns of mammillary and supramammillary label following the injection of an anterograde tracer in the dorsal hippocampus (Figure 9). The other provides the summary information, which was asked to be placed in its own figure (now Figure 13). We have also worked to improve the quality of the images. Finally, as recommended by the Editor, we have revised the final paragraph of the Discussion to make it clearer. As part of this process, we have tried to streamline the Discussion in order to limit repetitions.

**Reviews:**

**Reviewer: 1**

Comments to the Author

The revised version of the paper seems to addresses the reviewers’ concerns, though in some places the additional text is ragged and long-winded, and I lose the thread when reading it. The final uploaded version has all the figures (almost right) but no figure captions, so I will assume they are appropriate.

Minor points:

1. Page 26, discussion: “Although the distribution of frontal cells that project to these two diencephalic sites was more complex, it was again found that the populations remained distinct, often separated by laminar.” The sentence cannot end with an adjective.

We have exchanged “laminar” with “layers”

2. Fig. 11 D-F images are not mentioned in the text. I’m not sure what the point was of adding Fig. 11 and the text in “anterograde tracer injections in frontal cortices” pp 15-16. The Discussion about projection to MMMB from DP and IL on p. 19 doesn’t refer to the data and doesn’t really say anything, and I don't understand the point of the added text. It doesn’t help that I don’t have a figure caption for Fig. 11. The contrast should be enhanced in the images in D-E. I can hardly see the light labeling.

To address the main question in the study (whether there are collateral projections to nucleus reuniens and the mammillary bodies), we inevitably had to consider the frontal inputs to the mammillary bodies (see page 4 section 2). Two related issues emerged. These are the need to distinguish supramammillary from mammillary body inputs and the apparent inconsistencies in the present literature. For these reasons we included cases with anterograde tracer injections in frontal areas. While these injections do not directly answer the question of potential collateralization, they help to clarify wider, related questions on frontal inputs to the mammillary bodies. Most importantly, case 214#1 and Figure 12 clearly show that the specific cell label we see in the dorsal peduncular cortex originates from retrograde tracer uptake in the mammillary bodies.

We now refer to Figure 11 (now Figure 12) in the relevant part of the Discussion and we have added a reference to panels D-F at p. 16 where the relevance of the data is briefly explained.

The contrast for panels D-E has been enhanced, as requested.

3. The new text on Additional retrograde tracer injections (P. 25) needs editing for correct use of modifiers (note underlining of fixed words): confined injections also labelled layer V relatively densely; “labelling was considerably denser”

We have modified phrases such as ‘more dense’ in accordance with the comments of the Reviewer.

4. Disc p. 17 “collaterise” should be “collateralize”

We have changed the spelling in p.17 (also p.21 where the same mistake occurred).

5. The figure quality is improved but could still be better—more contrast enhance mainly would help.

We have enhanced contrast and brightness in the two figures. In Figure 7, panels A-E have increased contrast, which we believe improves image quality. Also in Figure 12, panels D-E has been modified so that the fibre labelling is more visible.

**Reviewer: 2**

Comments to the Author

The authors made significant improvements in the manuscript but some issues still need to be addressed.

Comments:

1. Although the expanded Figs 1 and 2 are helpful, it is still very difficult to separate each of the retrograde cases (or the patterns of labeling resulting from them) in that most of the findings are descriptive with only limited examples of actual patterns of labeling: schematics or micrographs. It certainly also does not help that cases are designated, for example, 207#2, 207#4 or 207#9, etc. What is very much needed for the retrograde cases is a Table listing each of the injections and their main subicular and cortical inputs – to thereby allow for comparisons across cases. This is particularly needed for cortical afferents as sites projecting to reuniens and MB are numerous and widely distributed.

Following these suggestions, we have added a Table that lists all the cases with combined retrograde tracer injections in multiple sites. The Table also shows whether cell label is present in the frontal cortices as well as in the subiculum. The list also gives the laminar and septo-temporal organization of any subicular cell label.

2. They indicate that it was possible to differentiate MB from SUM inputs on the basis and selective and differential brainstem afferents to each site: to MB from Gudden’s tegmental nucleus and to SUM from the laterodorsal tegmental nucleus. It is questionable if this is truly the case or if these criteria were actually used – no data is shown. For instance, as was indicated (p. 14) case 207#7 appears to significantly involve the SUM but virtually no labeled cells were observed in LDT. Unless substantiated, reference to Gudden’s and LDT this should be removed.

We did, as stated, analyse these cases for retrograde cell label in the laterodorsal tegmental nucleus in order to refine the evaluation of tracer uptake in the mammillary bodies and adjacent loci. However, it is not essential for us to include these data in the paper as it is only in one case (207#7) that we saw an apparent difference between this tegmental label and visual inspection of the CTB injection site. Furthermore, the labelling we observed in the laterodorsal tegmental nucleus was weak and scattered, which seems slightly out of step with the denser labelling reported in previous studies (e.g., Hayakawa et al, 1993). We have, therefore, removed reference to the laterodorsal tegmental nucleus label in the text.

3. It was important to make anterograde injections (3 injections) in the subiculum to in part determine whether the SUB projects to the SUM – which apparently it does not. However, no data is shown for this: schematics/micrographs for injection sites or terminal labeling. It would be valuable to include the actual data. Also, what was the pattern of labeling in RE with SUB injections -- and did labeling extend to RH. Finally, if the subicular injections were layer specific this would significantly support their retrograde findings of deep SUB projections to RE and superficial ones to MB.

We have included two additional cases that we feel are particularly helpful in this regard. These cases (212#10 and 212#11) received injections of adenoassociated virus (AAV) coding for the GFP protein. In both cases the injections were centred in the dorsal subiculum. This virus shows very robust anterograde transport to the mammillary bodies that contrasts with very light label in the supramammillary nucleus (see new Figure 9).

These same additional cases both show dense anterograde labelling in nucleus reuniens. These cases also show labelled fibres in the paraxiphoid nucleus as well as the posterior hypothalamus ventral to reuniens, although this fibre innervation is clearly weaker than that in reuniens. The rhomboid nucleus also, receive fibres from the dorsal subiculum, but at a much lower density to that in nucleus reuniens. In contrast, denser anterograde label is present in the submedius nucleus of the thalamus. We have now added this information to the Results and the Discussion, as it is relevant to how we interpret those injections within diencephalic nuclei.

The three other cases (BDA and WGA injections described in the previous version of the paper) only showed only extremely limited fibre labelling in reuniens. It is not, however, possible to confirm whether this shortage of labelling was caused by a lack of uptake in the deepest subiculum cell layers.

4. The panels on the figures are nicely labeled (A, B, C, etc) but in the text of Results the figures are most often referred to as a whole (e.g., Fig 7) without specific reference to individual panels. This is particularly bothersome on pgs. 12 and 13 when referring to Fig. 9 – and not specifying the individual panels. Also it is best to avoid the use “former” and “latter” as was often done in the manuscript.

We agree that it was a mistake not to reference individual panels. We have now added references to individual panels in those cases where it is relevant (including Figures 7 and 9, as requested). If a figure reference does not refer to specific panels it is because all panels are relevant.

We have also edited the text to remove the designations ‘former’ and’ latter’ (e.g. on page 6) as we agree that this can be ambiguous. More specific terminology has been used.

5. They reported (p. 14) that they did not observe any retrogradely-labeled cells in the frontal cortex with case 207#9. But examination of the FB injection in this case (207#9) shows it to be essentially equivalent to that of case 208#9 -- which exhibited robust retrograde cell labeling throughout the prefrontal cortex (Fig. 9). What was the difference in the two injections? This needs to be addressed.

As mentioned, a single case (207#9) lacked frontal labelling although it showed clear retrograde transport to other brain areas. The injection site in nucleus reuniens did not seem to vary substantially from similar injections with robust frontal transport (e.g., case 208#9). We are loath to dwell on this difference as while it may reflect some presently unknown subtle difference in cortico-thalamic organization, we have only one case from which to draw conclusions.

6. The section of the Results on indusium griseum (IG) (p. 13) is confusing and difficult to follow. This is partly due to interspersing dorsal and ventral aspects of IG as well as other cortical labeling when describing IG. For instance, what does “In both cases” (first sentence of the 2nd para on p. 13) refer to. Or “For both cases” – first sentence of the last para on p. 13. See also pts. 1 and 4 above.

We have tried to improve the sections in two ways. First, we have separated the descriptions of cortical label and label in the IG. The sentence on retrosplenial label has been moved to the previous section, where other cortical label is presented. The sentence on secondary motor area and cingulate label has been deleted, since this information is already contained in the paragraph above. Also, the description of dorsal IG label caudal to the genu of the corpus callosum has been simplified, making it more straightforward. Second, we have added the case numbers (208#9; 209#3) in both sentences in order to specify that we are referring to these two cases.

7. Eight anterograde tracer injections were made in regions of the PFC (Fig. 3). In the first instance, Fig. 3 should appear before Fig. 11. Apparently, the main reason for including the anterograde cases was to show that DP injections gave rise to terminal labeling in medial MB and not in SUM and also that PFC injections that avoided IG produced sparse labeling in MB? Did the dorsal peduncular injections produce labeling in RE; this should at least be discussed. Additionally, the anterograde PFC injections would be expected to yield valuable information on PFC projections to the ventral midline thalamus (or to SUM) – possibly confirming the findings of previous reports. This data may not be important to include here, but the issue should be addressed.

To answer the specific question, dorsal peduncular, infralimbic, and cingulate injections all resulted in terminal label in nucleus reuniens. This information is briefly given in the text.

Likewise, we have added brief information on the prefrontal inputs to the ventral midline thalamus. This information helps to confirm previous reports.

In the present version of the paper Fig. 3 appears at page 9 in the beginning of the result sections and Fig. 12 (figure that was previously Fig. 11) appears at page 16 in the section on anterograde tracer injections.

8. The summary of the interconnections (bottom of Fig. 11) should probably be a separate figure.

The summary is now included in an independent figure (Fig. 13).

9. Would replace the word “frequently” in the Abstract with “most often”.

We have replaced the words accordingly.

In summary, the main findings of the report regarding differential subicular and PFC input to MB and RE are solid, convincing and important. The above comments are for the most part relatively minor with the possible exception of points 1 and 3.

------------------------

EJN: AUTHOR'S INFORMATION & CHECK-LIST

Author Guidelines link: http://onlinelibrary.wiley.com/journal/10.1111/(ISSN)1460-9568/homepage/ForAuthors.html

3r^d^ Editorial Decision	07/11/18

_____________________________________________________________________________________

Separate cortical and hippocampal cell populations target the rat nucleus reuniens and mammillary bodies 

Dear Dr. Mathiasen, 

Your revised manuscript has been re-evaluated by one of the external reviewers as well as by the Section Editor, Dr. Helen Barbas and ourselves.  We are pleased to inform you that  it will be accepted for publication in EJN pending a few tiny revisions. 

Please address the couple of minor points raised by the reviewer.  Please also include figure legends in the main text. The author contributions must include how every author was involved in either writing or reviewing paper.   

When finalised, please upload your complete revised manuscript onto the website as a Word (.doc or .docx), or .rtf file.  Please also ensure that a complete set of tables and figures is included as separate files, even if these have not changed from the originals.  At this stage it is necessary to provide high resolution figures.  Please see important instructions below. 

Please go into https://mc.manuscriptcentral.com/ejn - Author Centre - manuscripts with decisions where you will find a 'create a revision' link under 'actions'. We ask that you please indicate the way in which you have responded to the points raised by the Editors and Reviewers in a letter. Please upload this response letter as a separate Word (.doc) file using the file designation "Authors' Response to Reviewers" when uploading your manuscript files. Please DO NOT submit your revised manuscript as a new one.  Also, please note that only the  Author who submitted the original version of the manuscript should submit a revised version. 

If you are able to respond fully to the points raised, we shall be pleased to receive a revision of your paper within 30 days. 

Thank you for submitting your work to EJN. 

Best wishes, 

Paul Bolam & John Foxe 
co-Editors in Chief, EJN 

Reviews: 

Reviewer: 2 

Comments to the Author 
The authors did a superb job in their latest revision of the manuscript – addressing all my previous concerns. The addition of the new viral based anterograde tracing cases and the Table were very valuable.   

Only two things: p. 12, animals not animas, and Fig 9 would suggest rearranging the SUM arrows for as now depicted it could seem that the SUM arrows are pointing to the dense fiber labeling in MB.

_____________________________________________________________________________________

Authors’ Response

_____________________________________________________________________________________

Dear EJN,

Two small typing errors are corrected in the final ms (see notes in step 1). At p. 6 "and PHA-L injections" is deleted since PHA-L was only injected iontophoretical, not mechanical. At p.7 "immunohistochemical processing" is changed to "immunofluorescence and immunohistochemical processing". In both cases, these corrections clarifies what already appears from the result section. Also, we have changed the image for the graphical abstract to a low resolution version (because of the 100MB limit). The graphical abstract is the same as Fig 8 which we have uploaded in a better resolution. We would prefer if the image uploaded for Fig 8 could be used as the graphical abstract,

Thanks,

Mathias L Mathiasen
